# Behavioral decline in *Shank3*^*Δex4–22*^ mice during early adulthood parallels cerebellar granule cell glutamatergic synaptic changes

**DOI:** 10.1186/s13229-024-00628-y

**Published:** 2024-12-04

**Authors:** Rajaram Kshetri, James O. Beavers, Romana Hyde, Roseline Ewa, Amber Schwertman, Sarahi Porcayo, Ben D. Richardson

**Affiliations:** 1 Department of Pharmacology, Southern Illinois University – School of Medicine, Springfield, IL 62702 USA; 2 Department of Biological Sciences, University of Idaho, Moscow, ID 83844 USA

**Keywords:** Shank3, Autism spectrum disorder, Phelan-McDermid syndrome, Cerebellum, Glutamate receptor, AMPAR, Granule cell, Mouse behavior

## Abstract

**Background:**

*SHANK3*, a gene encoding a synaptic scaffolding protein, is implicated in autism spectrum disorder (ASD) and is disrupted in Phelan-McDermid syndrome (PMS). Despite evidence of regression or worsening of ASD-like symptoms in individuals with PMS, the underlying mechanisms remain unclear. Although Shank3 is highly expressed in the cerebellar cortical granule cells, its role in cerebellar function and contribution to behavioral deficits in ASD models are unknown. This study investigates behavioral changes and cerebellar synaptic alterations in *Shank3*^*Δex4–22*^ mice at two developmental stages.

**Methods:**

*Shank3*^*Δex4–22*^ wildtype, heterozygous, and homozygous knockout mice lacking exons 4–22 (all functional isoforms) were subjected to a behavioral battery in both juvenile (5–7 weeks old) and adult (3–5 months old) mouse cohorts of both sexes. Immunostaining was used to show the expression of Shank3 in the cerebellar cortex. Spontaneous excitatory postsynaptic currents (sEPSCs) from cerebellar granule cells (CGCs) were recorded by whole-cell patch-clamp electrophysiology.

**Results:**

Deletion of *Shank3* caused deficits in motor function, heightened anxiety, and repetitive behaviors. These genotype-dependent behavioral alterations were more prominent in adult mice than in juveniles. Reduced social preference was only identified in adult *Shank3*^*Δex4–22*^ knockout male mice, while self-grooming was uniquely elevated in males across both age groups. Heterozygous mice showed little to no changes in behavioral phenotypes in most behavioral tests. Immunofluorescence staining indicated the presence of Shank3 predominantly in the dendrite-containing rosette-like structures in CGCs, colocalizing with presynaptic markers of glutamatergic mossy fiber. Electrophysiological findings identified a parallel relationship between the age-related exacerbation of behavioral impairments and the enhancement of sEPSC amplitude in CGCs.

**Limitations:**

Other behavioral tests of muscle strength (grip strength test), memory (Barnes/water maze), and communication (ultrasonic vocalization), were not performed. Further study is necessary to elucidate how Shank3 modulates synaptic function at the mossy fiber-granule cell synapse in the cerebellum and whether these changes shape the behavioral phenotype.

**Conclusions:**

Our findings reveal an age-related exacerbation of behavioral impairments in *Shank3*^*Δex4–22*^ mutant mice. These results suggest that Shank3 may alter the function of glutamatergic receptors at the mossy fiber-cerebellar granule cell synapse as a potential mechanism causing cerebellar disruption in ASD.

**Supplementary Information:**

The online version contains supplementary material available at 10.1186/s13229-024-00628-y.

## Background

It is estimated that one in every 36 children in the United States is diagnosed with autism spectrum disorder (ASD) [1]. Although the precise underlying causes and neurological mechanisms of ASD are poorly understood and likely diverse, disruption of multiple genes is linked to ASD [2–7]. Of the genes most strongly associated with ASD in recent exome sequencing studies [6–8], the *SHANK3* gene is consistently identified and has long been considered a monogenic cause of ASD [9, 10]. Haploinsufficiency of *SHANK3*, arising from mutations or deletions [4, 11–14] that disrupt SHANK3 protein expression or function is identified in a notable proportion (0.5-2%) of individuals with ASD and is the cause of Phelan-McDermid syndrome (PMS, 22*q*13.3 deletion) [15–18]. PMS is characterized by a high prevalence of ASD (84%) or intellectual disability (77%) [11, 19]. Although clinical data suggest that many individuals with PMS undergo a delayed regression or worsening of ASD-like behaviors [20–25], the timing and extent to which animal models with *Shank3* mutations/deletions recapitulate this regression is still emerging [26–29].

*Shank* genes (*Shank1*, *2*, and *3*) encode a family of multi-domain-containing proteins that serve as synaptic scaffolding and regulatory proteins for NDMA, AMPA, and metabotropic (mGluR) glutamate receptors at postsynaptic densities [13, 30–32]. Due to splice variants of its 22 exons, the Shank3 protein has six isoforms (A-F) that are uniquely expressed in particular brain regions [13, 33], with mouse behavioral phenotype and changes in neuronal function varying based on the *Shank3* exons/isoforms deleted [13, 34–47]. Unfortunately, the deletion of Shank3 isoforms from specific cell types or brain areas in rodents, like forebrain and striatum [41] has not yet led to a clear understanding of where in the brain Shank3 is critical for shaping all behavioral domains affected by *Shank3* disruption.

One area of the brain in which Shank3 expression steadily increases [33] during development and through adulthood is the cerebellar cortex, particularly Shank3C/D isoforms in cerebellar granule cells (CGCs) [33, 44–46, 48], where glutamate receptors (AMPAR, NMDAR, and mGluR) function is important for both development [49, 50] and synaptic processing by mature CGCs [51–56]. Despite the role of Shank3 in the regulation of glutamate receptors and its expression in developing and adult CGCs, only one study has evaluated the role of Shank3 in the cerebellum, identifying deficits in cerebellar learning in heterozygous *Shank3*^*Δex21*^ mice [40]. Given the established link between cerebellar dysfunction and ASD [10, 57–64] and the high-level expression of ASD-linked genes in the cerebellum [7, 65], understanding the role of Shank3C/D isoforms in even basal synaptic function of CGCs may be an important component of conceptualizing PMS and ASD.

Although the cerebellum is well-described for its role in motor control and motor learning [66, 67], the crystalline-like cerebellar cortex shapes the activity of the cerebellum afferents that project to both motor and non-motor brain regions [68–71]. These mono- and polysynaptic connections to non-motor brain areas are diverse (e.g. hypothalamus, ventral tegmental area, and hippocampus) and, along with functional studies, indicate cerebellar involvement in cognitive, affective, reward, motivation, and sensory processing [60, 68, 72–79]. This expanding list of brain areas and functions that involve the cerebellum establishes a rich network of interactions by which cerebellar dysfunction may impact a broad array of neural functions, processes, and behaviors.

Given that individuals with PMS can undergo behavioral regression during childhood and adolescence that continues into early adulthood [20–25], leveraging an ideal animal model displaying similar regression may be key to identifying brain regional and molecular mechanisms that drive this regression. Preferred assessment of animal model behavior should account for wildtype/heterozygous/homozygous genotypes, both sexes, age ranges analogous to key human age ranges, circadian effects (light vs. dark phase), and behavior across a range of domains. Accounting for these factors and to bridge gaps in current literature assessing behavioral regression in the absence of some or all Shank3 isoforms [26–29], we assessed the behavioral phenotype of male and female *Shank3*^*Δex4–22*^ mice in two separate age cohorts during the dark phase. Then, to determine whether cerebellar dysfunction corresponds to the development of the behavioral phenotype across early adulthood, we evaluated the expression of Shank3 at CGC synapses and differences in spontaneous excitatory postsynaptic currents in wildtype and homozygous *Shank3*^*Δex4–22*^ knockout mice corresponding to both age cohorts and sexes.

## Methods

### Animals

All animal procedures were performed per protocols approved by the Institutional Animal Care and Use Committee at Southern Illinois University – School of Medicine or the University of Idaho. C57BL/6J mice (JAX stock no.: 000664) and *Shank3*^*Δex4–22*^ mice initially described by Drapeau et al., 2018 [47] (JAX stock no.: 032169) and were acquired from Jackson Laboratories and/or bred in-house to generate animals used in all experiments. *Shank3*^*Δex4–22*^ mice were maintained on a C57BL/6NJ genetic background as provided by the vendor [42, 47]. These mice lacking exons 4–22 of the *Shank3* gene lack expression of all major isoforms A-F that are differentially expressed throughout the brain [33, 42, 47]. A heterozygous/heterozygous breeding strategy was employed to generate *Shank3*^*Δex4–22*^ wildtype (+/+, WT), heterozygous (-/+, Het), and homozygous knockout (-/-, KO) mice used for all behavioral and electrophysiology experiments. Offspring genotypes were determined through Transnetyx (Cordova, TN) using ear punch or tail biopsies. All mice were group housed with one to three other mice on a reversed (12 h/12hr) light-dark cycle with *ad libitum* access to food and water.

### Behavioral battery

Mice of both sexes representing all three *Shank3*^*Δex4–22*^ genotypes were randomly tested on a behavioral battery to assess motor, anxiety, sociability, repetitive, and memory behaviors described below with the number of assays and order of completion for each cohort chosen randomly. Some mice did not always complete all assays. Separate cohorts of mice were evaluated on the behavioral battery at either a juvenile (5–7 weeks) or young adult age (3–4 months) with no mice exposed to the same assay more than once in their lifetime. All behavioral testing was completed in low red-light conditions (15–20 lx) during the dark phase. Video tracking and automated analysis (Noldus EthovisionXT v17.5) of animal behavior in the open field, elevated zero maze, Y-maze, and sociability assays were used to evaluate animal location. For all other assays and manual scoring in an open field, experimenters were blinded to the genotype of all mice during testing and for manual analysis. Before each behavioral test, animals were habituated for 30 min in the testing room and each apparatus was thoroughly cleaned to reduce the impact of odor cues that may interfere with behavior. Statistical results for all behavioral data analyses are presented in Supplementary Material 1.

### Open field

Mice were evaluated in an open field (40 × 40 cm box) for a total of 30 min to assess gross motor function, locomotor behavior, and other stereotypical behavioral patterns. Mice were observed in the open field to determine total voluntary distance traveled, time within the center (20 × 20 cm) region equidistant from all edges, total entries into the center region, freezing time, total amount of time spent grooming, maximal speed, and total number of fecal boli deposited. To determine mouse location within the arena, the center point of the body was used.

### Elevated zero maze

The elevated zero maze comprises a circular (5 cm wide) track with an inner diameter of 40 cm that is elevated 60 cm above the ground. The annulus is divided into four equal quadrants, wherein two opposing quadrants are left open and the remaining two alternate quadrants are enclosed by 40 cm high opaque walls. The mice were placed in an open arm and allowed to freely explore the maze with various parameters assessed, such as the duration spent in the open quadrants and the number of entries into the open quadrants. Mice that spend more time in the closed quadrants and exhibit fewer entries into the open quadrants are generally considered to have higher levels of anxiety, and vice versa. To determine mouse location within the arena, the center point of the body was used.

### Rotarod

To evaluate motor and vestibular function, mice were evaluated to determine the duration of time they were able to remain on a rotating rod (3.17 cm diameter, IITC Life Sciences, Inc., Woodland Hills, CA, USA) that was continuously accelerating from 4 to 40 RPM over 5 min. Each mouse was tested on the rotarod for three trials per day for two consecutive days (six total trials), with a 10-minute intertrial interval. For each trial, the time at which a mouse remained attached to the rotating rod for one complete rotation and the time at which the mouse fell from the rod to the landing platform were recorded. A reduced latency to fall will indicate motor deficits and a lack of improvement in subsequent trials indicates reduced motor learning ability [46, 47].

### Beam balance

To evaluate fine motor coordination and balance that might not be detected by other tests of more gross motor function, mice were placed at one end of a horizontal flat beam (1 m long, 12 mm–6 mm wide) and allowed to walk across the beam to a dark goal box (20 cm cube). First, mice were trained for two consecutive days, consisting of three trials on both the 12 mm and 6 mm beam with each trial for a given beam separated by a 1-minute rest period in addition to a 10-minute rest period between each beam. Subsequently, each mouse’s performance was evaluated on both beams on the third day when were tested on each beam twice. Performance during the test day was analyzed to determine the time to reach the dark box and the number of paw slips while traversing the beam [47, 80]. Beam crossing time and total number of foot slips are an average of the two test trials.

### Gait analysis

Footprint analysis was used to quantify potential variations in gait as an indicator of fine motor functional capacity. Mice were first trained to traverse a corridor runway (1 m long x 5 cm wide) lined with white standard electrocardiograph paper with a dark goal box placed at the opposite end of the corridor. After three training trials, the mouse’s paws were coated with nontoxic blue (front paws) or red (hind paws) paint to record paw placement on two consecutive runs. Stride length and width of the forelimbs and hindlimbs were determined by measuring the respective distances from the paw center as shown in Fig. 4A for the second test trial [80, 81].

### Marble burying

Burying of small objects is a naturalistic behavior in mice with changes in the engagement in this behavior proposed to be related to anxiety-like, repetitive, compulsive, and/or perseverative behavior [82, 83]. First, each mouse was placed in an empty clean standard mouse cage (17 × 28 × 13 cm) with 3 cm of bedding for 5 min. Then, the mouse was removed and sixteen marbles were placed in the cage on top of the bedding. The mouse was then placed back in the cage and their activity was recorded for 30 min. The video record was evaluated to determine the number of marbles that are at least 50% covered by bedding at each 5-minute time interval.

### Y-Maze spatial working memory

To assess short-term working memory, mice were placed in the center of a “Y”-shaped maze composed of three 35 cm long arms (5 cm wide) extending out from a central point at 120° from one another with 20 cm tall walls. Mice were allowed to freely explore the novel Y-maze environment for 10 minutes with the center point of the mouse’s body crossing into the arm considered as an entry. The total number of arm entries recorded to assess exploratory behavior and the percentage of alternate arm entries into the least recently visited arm (as opposed to the most recently visited arm) was taken as a measure of short-term working memory function [84].

### Three-chamber sociability

To evaluate social behavior, mice were evaluated in a four-phase protocol within an arena (40.5 cm wide, 60 cm long, and 22 cm high) that was divided into three equal-sized chambers with openings in the dividers to allow mice to travel into each chamber. The center chamber of the arena was empty and the two chambers at opposing ends each contained one circular barred cage in the center of the chamber. The sociability assay protocol consisted of four 5-minute-long phases with the mouse placed back into the center chamber with doors between each chamber closed in between each phase. First, the test mouse was placed in the center chamber of the apparatus with the two empty cages present and the mouse was allowed to freely explore all three chambers. In the second pre-test phase, each mouse was allowed to explore the entire arena with two identical inanimate objects inside each cage. In the third phase, the mouse was allowed to freely explore the arena with one of the inanimate objects replaced with an unfamiliar wildtype mouse (similar age and same sex) and a novel non-social stimulus (inanimate object) contained within the other cage. Finally, in phase four, the non-social inanimate object was replaced with another unfamiliar wildtype mouse of a similar age and same sex to serve as a novel social stimulus, then the test mouse was allowed to interact with both familiar and unfamiliar mice. The amount of time the mouse spent within 2 cm of the cage containing the social stimulus (T_S_), non-social stimulus (T_NS_), familiar mouse (T_F_), and novel mouse (T_N_) were quantified and used to calculate the social preference index (I_SP_ = (T_S_ − T_NS_)/(T_S_ + T_NS_)) or social novelty index (I_SN_ = (T_N_ – T_F_)/(T_N_ + T_F_)) [85]. During the sociability assay, we observed that eight mice (2 WT, 6 KO) displayed a strong bias toward one side of the chamber that never entered one side of the sociability chamber. This complete absence of time spent on one side of the chamber made index calculations problematic and were therefore not included in the analysis of social preference and social novelty preference behavioral data.

### Immunohistochemistry

For immunohistochemical analysis of Shank3 distribution in the cerebellar cortex, male C57BL/6J mice were anesthetized using isoflurane (3–5%) and then transcardially perfused with 1X phosphate-buffered saline (PBS) followed by 4% formaldehyde diluted in 1X PBS. Brains were then removed and post-fixed for 48–72 h in 4% formaldehyde followed by placement into 30% sucrose in 1X PBS for at least 24 h before sectioning. Sagittal 40 μm thick slices of the cerebellum were prepared on a cryostat. Sagittal sections of the cerebellar vermis were then washed in 1X PBS and then permeabilized and blocked in 95% methanol 5% acetic acid for 10 min followed by IHC/ICC Blocking Buffer (eBiosciences) with 0.5% triton-X 100 for 1 h. To block endogenous IgG and reduce labeling by mouse primary antibodies, all slices were subject to a second blocking step of polyclonal goat F(ab) anti-mouse IgG (1:100; ab6668, Abcam) diluted in 1X PBS. Tissue sections were then incubated at room temperature for 4 h in primary antibodies that included monoclonal mouse IgG1 anti-VGlut1 (1:500; Neuromab/Ab Inc., #75 − 066), polyclonal chicken IgG anti-VGlut2 (1:500; Synaptic Systems, #135–416), and polyclonal rabbit anti-Shank3 (1:1000; Alomone, APZ-013). Primary antibodies were then labeled for 2 h at room temperature with secondary antibodies conjugated to fluorescent tags diluted with 1X PBS that included goat anti-chicken AlexaFluor488 (1:500; Invitrogen, A11039), goat anti-mouse IgG1 AlexaFluor568 (1:500; Invitrogen, A21124), and donkey anti-rabbit AlexaFluor647 (1:500; Invitrogen, A31573). Immediately after immunolabeling, tissue sections were washed and transferred to glass slides and mounted with Prolong Gold (Invitrogen). Multi-plane confocal images were acquired using 4X, 20X, and 60X objective magnification with comparable image settings on a Nikon Spinning Disk Confocal Microscope.

To determine the degree of colocalization postsynaptic Shank3 in cerebellar granule cell (CGC) dendrites with presynaptic VGlut1- and VGlut2-positive mossy fibers (MFs), multi-color single plane confocal images were evaluated using the Mander’s Coefficient. Ranging from 0 to 1, the Mander’s Coefficient indicates the proportion of the colocalizing pixels in each color channel, which is less sensitive to background noise than Pearson’s R [86]. Specifically, the Mander’s Coefficient using the auto-threshold regression of the target channel was used to assess colocalization within manually selected regions of interest (ROI) based on the profile of presynaptic MF (VGlut1 or VGlut2) terminals (see Fig. 7I) using ImageJ/Fiji (NIH). All ROIs were pooled for each of two images of the internal granule cell layer in non-unipolar brush cell expressing regions, and the pooled data from each confocal image (*n* = 10) were analyzed per animal (*N* = 5).

### Electrophysiology

To prepare acute brain slices for recording from cerebellar granule cells, brains from juvenile (6–8 weeks) or young adult (3–6 months) *Shank3*^*Δex4–22*^ wildtype, heterozygous, and homozygous knockout mice were rapidly removed and placed in ice-cold sucrose slicing solution. This solution contained the following components (in mM): 2.5 KCl, 0.5 CaCl_2_, 4 MgCl_2_, 1.25 NaH_2_PO_4_, 24 NaHCO_3_, 25 glucose and 230 sucrose. The brain was then mounted to a holder and encased in agar and sliced parasagittally (250 μm) using a Compresstome VF-200 (Precisionary Instruments). The cerebellar slices were then transferred to a recovery solution that included the following components (in mM): 85 NaCl, 2.5 KCl, 0.5 CaCl_2_, 4 MgCl_2_, 1.25 NaH2PO_4_, 24 NaHCO_3_, 25 glucose, and 75 sucrose, maintained at 32 °C [87]. After 30 min of recovery, cerebellar slices were transferred to room temperature artificial cerebral spinal fluid (ACSF) containing (in mM): 124 NaCl, 26 NaHCO_3_, 1 NaH_2_PO_4_, 2.5 KCl, 2 MgCl_2_, 10 D-glucose, 2.5 CaCl_2_. All solutions were saturated with 95% O_2_ and 5% CO_2_, had a pH of 7.3–7.4 and osmolarity of 300–310 mOsm. Slices were transferred to a custom recording chamber on an upright Olympus BX51WI microscope and cerebellar granule cells in the internal granule cell layer in lobules 4–5 were visualized with a 60X water-immersion objective using infrared differential interference contrast. ACSF was continuously perfused into the chamber at the rate of 3–5 ml/min maintained at 32–34 ⁰C.

Whole-cell voltage-clamp recordings of visually identified CGCs were made using borosilicate patch pipettes (1.5 mm OD/0.86 mm ID) pulled with a P-1000 micropipette puller (Sutter Instruments) to have a tip resistance of (5–8 MΩ) when filled with CsCl-based internal solution (E_Cl_ = 0 mV) that contained (in mM): 130 CsCl, 4 NaCl, 0.5 CaCl_2_, 10 HEPES, 5 EGTA, 4 Mg-ATP, 0.5 Na-GTP, and 5 QX314 with pH adjusted to 7.2–7.3 with CsOH and an osmolarity of 280–290 mOsm [88, 89]. Whole-cell patch-clamp recordings were acquired with a Multiclamp 700B amplifier (Molecular Devices) and sampled at 20 kHz (10 kHz low pass filter) with a Digidata 1440 A (Molecular Devices). Following the formation of a gigaseal (> 1GΩ), the whole-cell configuration was produced by the application of rapid negative pressure to the pipette. Whole-cell membrane properties were determined by applying a 10-mV hyperpolarizing voltage step from the initial holding potential (-60 mV) in voltage-clamp mode. Whole cell recordings from CGCs had a series resistance of 20 ± 5 MΩ and recordings with variation in series resistance of greater than 20% throughout the recording were discarded. To isolate spontaneous excitatory postsynaptic currents (sEPSCs), CGCs were voltage-clamped at -60 mV and the GABA_A_ receptor antagonist, gabazine (10 µM; Tocris Bioscience) was present in the ACSF. Inward transient sEPSCs with a fast rise and exponential decay were analyzed over a 3–5 min period with Easy Electrophysiology Software (v2.6.0) by first-pass automatic threshold detection followed by manual inspection of events. All events from each CGC were used to construct a cumulative distribution histogram (Fig. 8C-F) for amplitude (1 pA bin size) or inter-event interval (IEI, 100 ms bin size). Event amplitude and IEI were averaged for each cell to generate group averages and for statistical comparisons between genotypes (Fig. 8C-F inset). Individual sEPSC amplitude histograms (5 pA bin) were constructed for each CGC and normalized to the total number of events. To reduce the impact of CGCs with high sEPSC frequencies across recordings, these normalized histograms created for each CGC were then averaged across groups (Fig. 8G) and fit with a Gaussian function (Fig. 8H).

### Statistical analysis

Mice of all three *Shank3*^*Δex4–22*^ genotypes and both sexes at two separate age groups (juvenile and adult) were evaluated in all behavioral assays and electrophysiology experiments with no mice evaluated at more than one age. Automated and manual determination of dependent variable values in EthoVision XT 17.5 were analyzed using SPSS 29 (IBM) and Igor Pro 8 (Wavemetrics). For comparison of group effects on dependent variables, a 3-way ANOVA (genotype, age, sex) or 3-way repeated measures ANOVA (MANOVA) were used for data with equal variance based on the median (Levene’s Test). Bonferroni correction for multiple comparison post-hoc tests on the estimated marginal means was used for pairwise comparisons to identify differences between genotypes with different ages and sexes when 3-way ANOVAs indicated significant main effects or interactions for those terms with genotype. For all behavioral assays, data are shown separated by genotype and age with additional separation of data by sex. When Mauchly’s test of sphericity was significant for MANOVAs, the Huynh-Feldt tests were used to determine time effects (open field, rotarod). For data without equal variance (Levene’s Test: *p* < 0.05; Box’s Test: *p* < 0.001), nonparametric Kruskal Wallis H tests were used to identify significant genotype effects within ages and sexes at each age since SPSS does not allow for multiple independent variables to be included in Kruskal Wallis H test. Mean colocalization values from each confocal image were compared to determine Shank3 expression differences between MF terminal types with an independent samples t-test. A t-test was used for the comparison of average synaptic event amplitudes, interevent intervals, and percentages of events within each 5 pA histogram bin between wildtype and knockout mice within age groups. All data values are reported as mean ± standard error (SEM) with individual markers representing the value for each observation, which is the animal (N) for all behavioral assays, the image (n) for confocal analysis, and the cell (n) for electrophysiology assays. Statistical results for all behavioral data analyses are presented in Supplementary Material 1.

## Results

### Anxiety-like behavior increases with age in the absence of Shank3

**Fig. 1:**
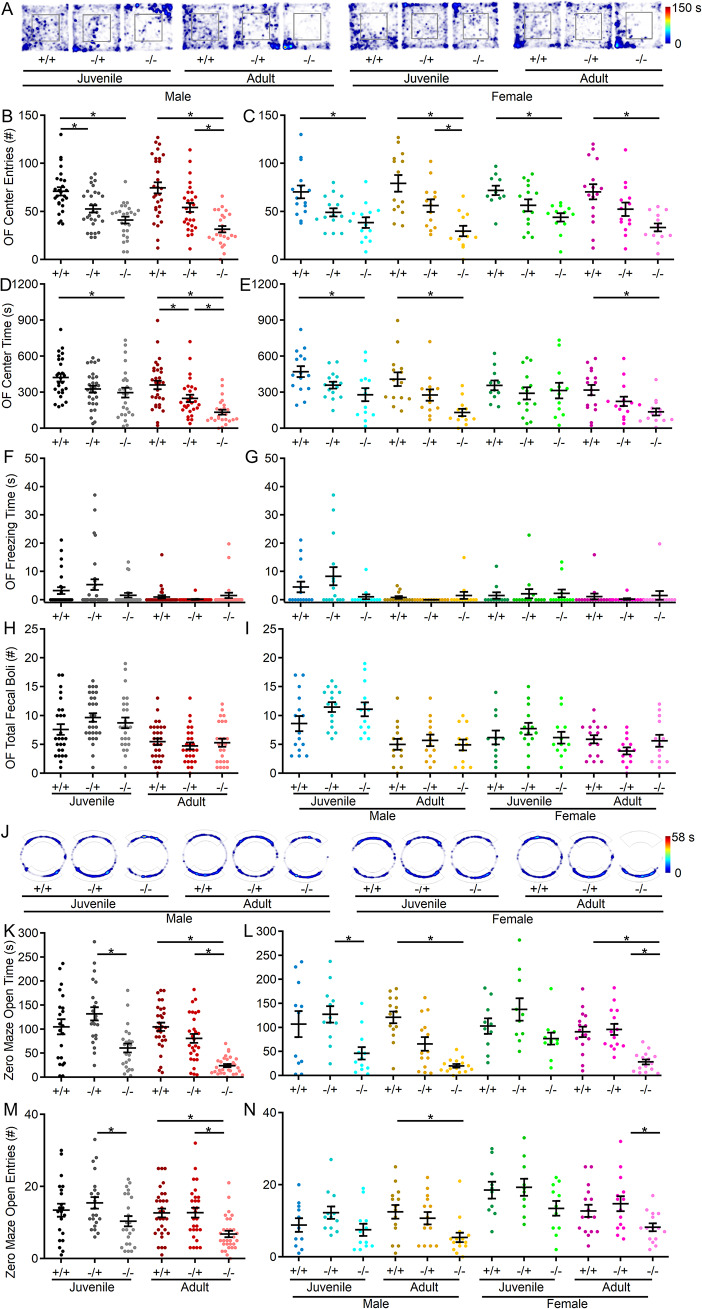
S*hank3*^*Δex4–22*^ KO mice display greater levels of anxiety with age. (**A**) Representative heatmaps of time spent in each area of the open field arena for one mouse of each genotype, age, and sex. (**B-I**) Individual (circles) and mean ± SEM (black bars) of the number of center entries (**B**,** C**), total open field center time (**D**,** E**), total freezing duration during open field exploration (**F**, **G**), and total number of fecal boli at the end of open field exploration (**H**,** I**) for each genotype at both ages (**B**,** D**,** F**, and **H**) and further separated by sex (**C**,** E**,** G**, and **I**). (**J**) Representative heatmaps of time spent in each area of the zero maze for one mouse of each genotype, age, and sex. (**K-N**) Individual animal (circles) and group mean ± SEM (black bars) of total open arm time (**K**,** L**) and the number of open arm entries (**M**,** N**) in the elevated zero maze for each genotype at both ages (**K**,** M**) and further separated by sex (**L**,** N**). For **A** and **J**, the color scale bar at right applies to all heatmaps in the corresponding assay. *N* = 22–30 mice/group for each genotype at each age and *N* = 10–16 mice/group for each sex within each genotype at each age. *p < 0.05 for post-hoc test between genotypes with a Bonferroni correction

To investigate anxiety-like behavior in *Shank3*^*Δex4–22*^ mice, the open field and elevated zero maze tests were conducted. In the open field (Fig. 1A), *Shank3*^*Δex4–22*^ knockout mice entered the center area less frequently at both ages compared to wildtype counterparts. This effect was observed in both males and females (Fig. 1B, C). Except for the juvenile knockout females, the time spent at the center of the open field was also reduced in all other *Shank3*^*Δex4–22*^ knockout groups (Fig. 1D, E). Although heterozygous mice displayed increased anxiety-like behavior at both younger (Fig. 1B) and adult time points (Fig. 1D) in pooled open field data, this effect disappeared when the data were separated by sex (Fig. 1C, E). No genotype effects were detected in total freezing duration (Fig. 1F, G) or the number of fecal boli deposited (Fig. 1H, I) at the end of the session. Although a significant interaction between age and genotype was not detected in the open field center area measures, the elevated zero maze was used as an alternate more sensitive measure of anxiety-like behavior (Fig. 1J). In the zero maze, adult *Shank3*^*Δex4–22*^ knockout mice spent less time in open arms (Fig. 1K, L) and entered open arms less often (Fig. 1M, N) compared to *Shank3*^*Δex4–22*^ wildtype and heterozygous mice. There was also a significant interaction between age and genotype corresponding to an absence of significant difference between zero maze open arm time between *Shank3*^*Δex4–22*^ wildtype and knockout juvenile mice. The increased avoidance of the open/exposed areas in both assays is indicative of heightened anxiety-like behavior with reduced Shank3 expression that escalates during the juvenile to adult transition.

### Reduced locomotor activity in *Shank3*^*Δex4–22*^ knockout mice is consistent throughout early maturity

**Fig. 2:**
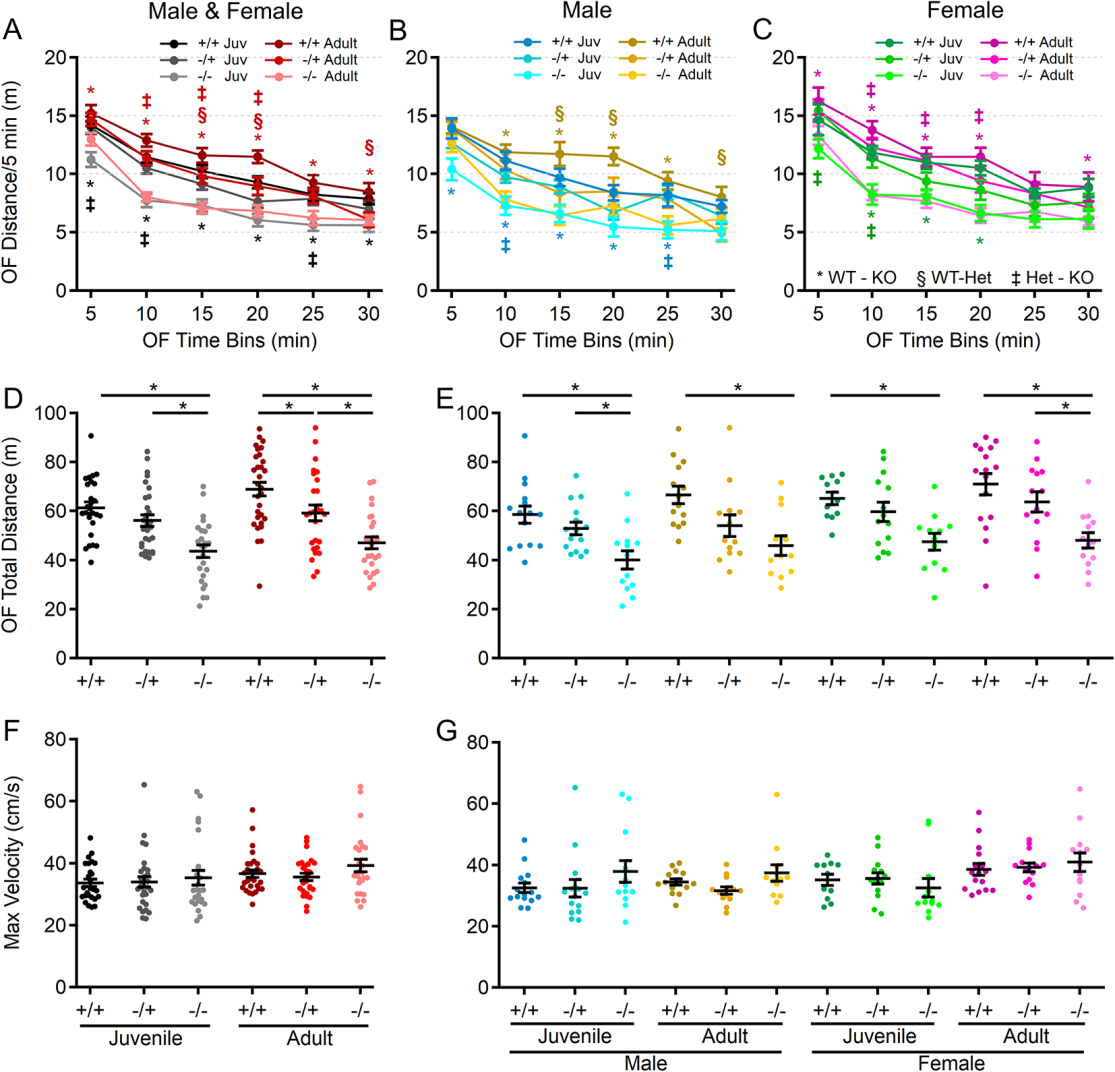
Juvenile and adult *Shank3*^*Δex4–22*^ KO mice display reduced exploratory and locomotion behavior. (**A-C**) Mean ± SEM of the total distance moved within each 5 min period of open field exploration for each genotype at both ages (**A**) and further separated into males (**B**) and females (**C**) at each age. (**D-G**) Individual animal (circles) and group mean ± SEM (black bars) of the total cumulative distance moved (**D**,** E**) and maximal linear movement velocity detected (**F**,** G**) during open field exploration for each genotype at both ages (**D**,** F**) and further separated by sex (**E**,** G**). *N* = 22–30 mice/group for each genotype at each age and *N* = 10–16 mice/group for each sex within each genotype at each age. In open field distance-time plots (**A-C**), symbols correspond to *p* < 0.05 in post-hoc comparison of genotypes within age and/or sex: * WT-KO, ‡ Het-KO, and § WT-Het, while *p < 0.05 in scatter dot-mean plots (**D-G**) in post-hoc comparisons between genotypes, all with a Bonferroni correction

Spontaneous locomotion in the open field was evaluated as an indicator of gross motor ability, exploratory behavior, and basal spontaneous activity (Fig. 2). Analysis revealed main effects of genotype, age, and sex, but no significant interactions among these variables. At both ages and in both sexes, *Shank3*^*Δex4–22*^ knockout mice demonstrated reduced spontaneous locomotion relative to wildtype mice (Fig. 2A-E), which persisted throughout the 30-minute session (Fig. 2A-C). Despite the lack of significant interaction between genotype and age, the difference in total distance moved in the open field between *Shank3*^*Δex4–22*^ wildtype and knockout mice was greater in the adult group compared to the juvenile group. There was a similar age-dependent shift in the adult heterozygous *Shank3*^*Δex4–22*^ mice which also significantly differed from wildtype mice in total distance moved (Fig. 2D).

### Motor performance declines with age in the absence of Shank3

**Fig. 3:**
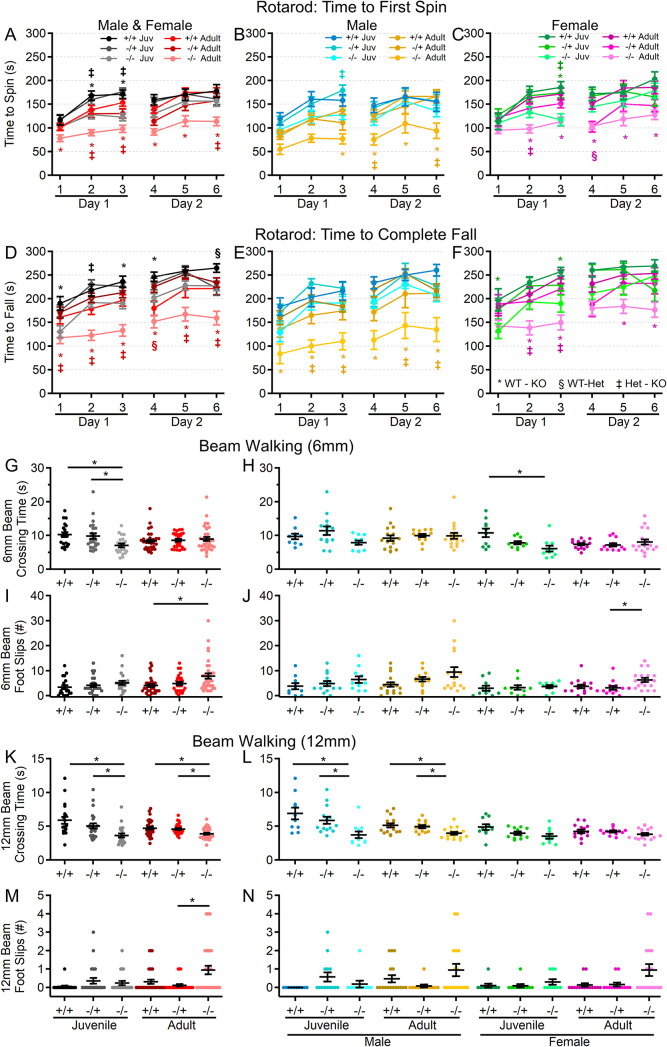
*Shank3*^*Δex4–22*^ KO mice develop motor function deficits with age. (**A-F**) Mean ± SEM of the time until the mouse rotates completely around the rotarod (**A-C**) or falls to the landing platform (**D-F**) for three subsequent accelerating rotarod tests (4–40 RPM, 5 min) repeated over two days total for each genotype at both ages (**A**,** D**) and further separated into males (**B**,** E**) and females (**C**,** F**) at each age. (**G-N**) Individual (circles) and mean ± SEM (black bars) of the time to cross (**G**,** H**,** K**, and **L**) and the number of left and right total foot slips (**I**,** J**,** M**, and **N**) on a 6 mm wide (**G-J**) and 12 mm wide (**K-N**) beam for each genotype at both ages (**G**,** I**,** K**, and **M**) and further separated by sex (**H**,** J**,** L**, and **N**). *N* = 20–35 mice/group for each genotype at each age and *N* = 10–18 mice/group for each sex within each genotype at each age. In rotarod time plots (**A-F**), symbols correspond to *p* < 0.05 in post-hoc comparison of genotypes within age and/or sex: * WT-KO, ‡ Het-KO, and § WT-Het, while *p < 0.05 in scatter dot-mean plots (**G-N**) in post-hoc comparisons between genotypes, all with a Bonferroni correction

Since motor performance is often affected in ASD and is one area in which PMS patients experience regression, motor performance of *Shank3*^*Δex4–22*^ mice was assessed in multiple assays, including the rotarod, beam walking, and gait analysis. As an indicator of gross motor ability, maximal linear velocity throughout the entire 30-minute open field session was assessed in all groups. Although there was no difference due to genotype (Fig. 2F, G), there was a significant main effect of age and sex. To provide a comprehensive view of motor and vestibular ability using the accelerating rotarod, the time (corresponding to rotation speed) an animal attached to the rod for one complete revolution (Fig. 3A-C) and the time when they completely fell off the rotarod to the landing platform (Fig. 3D-F) were both recorded. Latency to first spin times were shorter than the latency to fall times and juvenile mice generally had longer latencies than adult mice as did female mice relative to male mice at either age. Although both measures of rotarod performance identified a main effect of genotype, age, and sex, as well as significant age and sex interactions in later trials, the time to fall measure more robustly detected significant interactions between genotype and age (Fig. 3D-F) on rotarod performance. Specifically, adult male and female *Shank3*^*Δex4–22*^ knockout mice performed worse than wildtype and heterozygous mice in most trials. However, these deficits were only beginning to emerge in juvenile knockout mice in some trials (Fig. 3D-F). The overall rotarod performance of heterozygous mice was mildly reduced and most similar to that of wildtype mice (Fig. 3A-F).

In assessing motor coordination and balance by evaluating the ability to reliably traverse a narrow beam (Fig. 3G-N), both juvenile (on both the 6 mm and 12 mm beams) and adult (on the 12 mm beam) *Shank3*^*Δex4–22*^ knockout mice exhibited a reduced traversal time compared to wildtype mice when moving toward a closed goal box (Fig. 3G, H, K, L). However, only adult *Shank3*^*Δex4–22*^ knockout mice displayed an increased number of foot slips while crossing both the 6 mm (Fig. 3I, J) and 12 mm wide beams (Fig. 3M, N), which is in line with similar age-specific deficits in rotarod performance. The performance of heterozygous mice was comparable to that of wildtype mice.

**Fig. 4:**
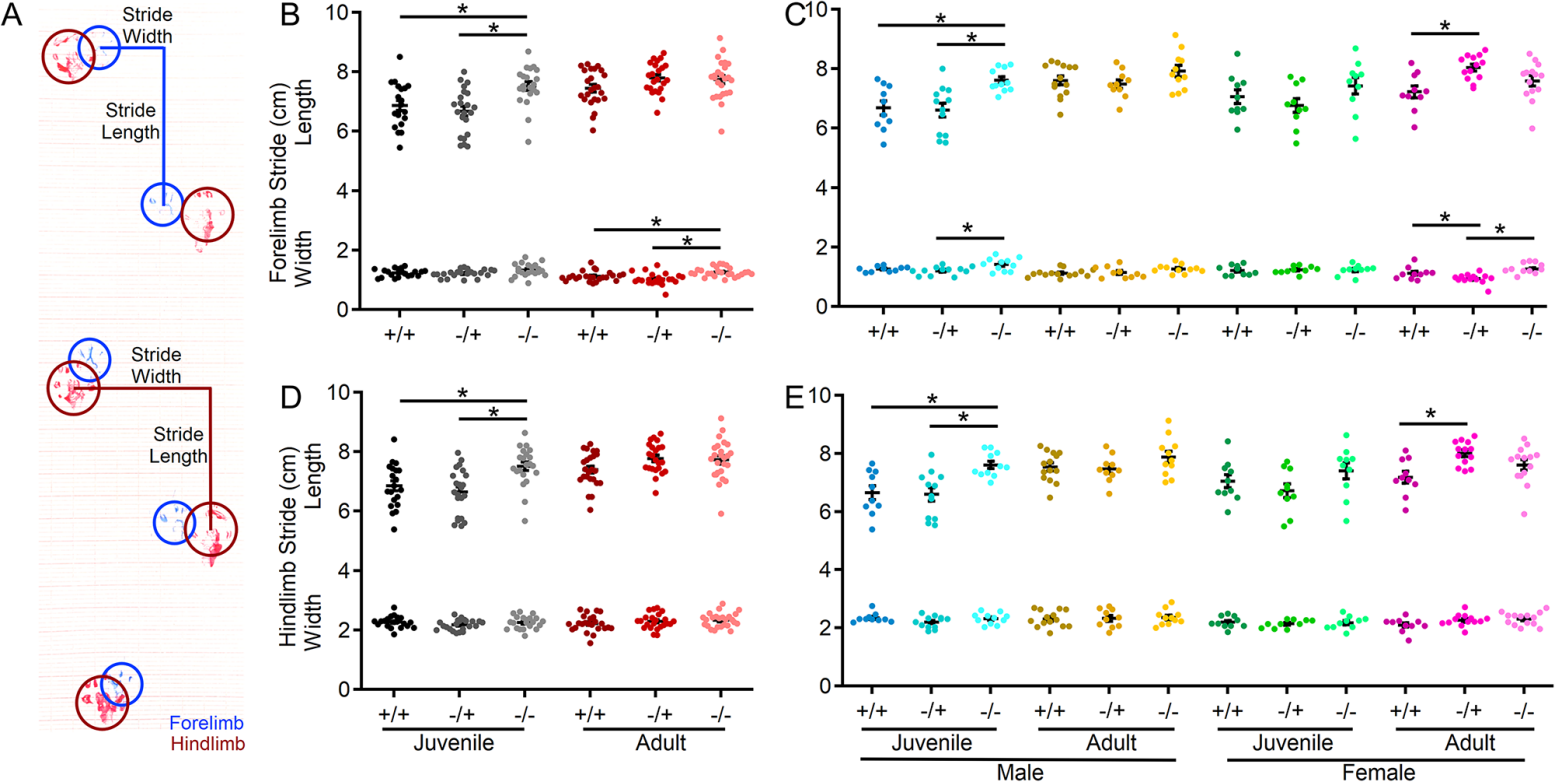
S*hank3*^*Δex4–22*^ KO mice develop an elongated stride length as juveniles. (**A**) Sample gait analysis raw data with location, stride length, and width of the forelimb identified in blue and hindlimb in red. (**B-E**) Individual (circles) and mean ± SEM (black bars) of the forelimb stride length and width (**B**,** C**) and the hindlimb stride length and width (**D**,** E**) for each genotype at both ages (**B**,** D**) and further separated by sex (**C**,** E**). *N* = 22–25 mice/group for each genotype at each age and *N* = 10–14 mice/group for each sex within each genotype at each age. *p < 0.05 for post-hoc test between genotypes with a Bonferroni correction

As a final assessment of motor function, gait analysis (Fig. 4A) was performed to assess changes in the forelimb and hindlimb stride length and width (Fig. 4). Except for hindlimb stride width, there were significant main effects of genotype and age on the remaining three parameters: forelimb stride length, forelimb stride width and hindlimb stride length (Fig. 4). Specifically, there was a significant elongation of both forelimb and hindlimb stride length in juvenile *Shank3*^*Δex4–22*^ knockout mice, which appeared to be more prominent in male mice. A similar trend was present in adult mice but may have been reduced due to variation in animal size by that age (Fig. 4B-E). Interestingly, only adult heterozygous female mice, but not homozygous knockouts, showed increased forelimb and hindlimb stride lengths, along with decreased forelimb stride width compared to wildtype mice (Fig. 4C, E). Together, these data support the idea that brain regions essential for balance and motor coordination, including the striatum and cerebellum—both of which express high levels of Shank3—may undergo escalating levels of disruption during early adulthood with the absence of Shank3.

### Increased repetitive self-grooming behavior in male *Shank3*^*Δex4–22*^ knockout mice

**Fig. 5:**
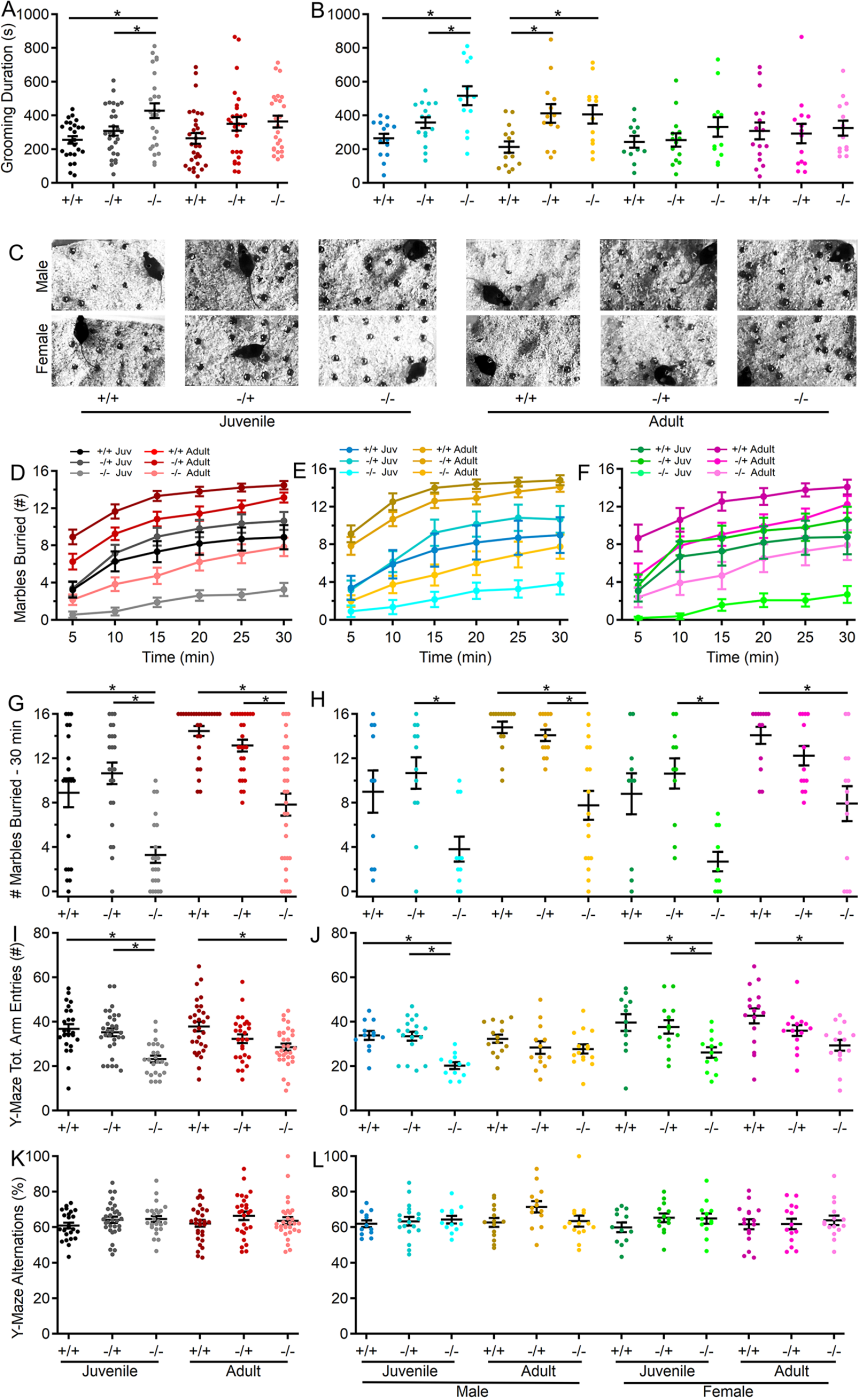
Loss of *Shank3* increases repetitive behavior in male mice and decreases exploratory behavior in juvenile mice of both sexes. (**A**,** B**) Individual (circles) and mean ± SEM (black bars) of the total duration of grooming time during open field exploration for each genotype at both ages (**A**) and further separated by sex (**B**). (**C**) Representative images of marble location after 30 min in the marble burying arena for one mouse of each genotype, age, and sex. (**D-F**) Mean ± SEM of the number of marbles buried after each 5 min period during the marble burying assay for each genotype at both ages (**D**) and further separated into males (**E**) and females (**F**) at each age. (**G**,** H**) Individual (circles) and mean ± SEM (black bars) number of marbles buried after 30 min for each genotype at both ages (**G**) and further separated by sex (**H**). (**I-L**) Individual animal (circles) and group mean ± SEM (black bars) of the total number of arm entries (**I**,** J**) and percent of alternations (**K**,** L**) in the Y-maze for each genotype at both ages (**I**,** K**) and further separated by sex (**J**,** L**). *N* = 20–31 mice/group for each genotype at each age and *N* = 10–18 mice/group for each sex within each genotype at each age*p < 0.05 for post-hoc test between genotypes with a Bonferroni correction

To assess repetitive and exploratory behaviors in the absence of Shank3, self-grooming activity in the open field and marble-burying activity were assessed in all groups. Unlike other behaviors tested, juvenile mice lacking both copies of *Shank3* displayed the most robust increase in self-grooming behavior relative to wildtype and heterozygous mice (Fig. 5A). Notably, this increase in repetitive self-grooming appeared to be restricted to male mice in both age groups (Fig. 5B). Furthermore, adult heterozygous males showed more grooming compared to wildtype controls. A significant genotype-sex interaction was observed, indicating sex-specific effects on grooming behavior in *Shank3*^*ex4–22*^ knockout mice.

### Reduced marble burying and exploratory behavior is pronounced in juvenile *Shank3*^*Δex4–22*^ knockout mice

The marble burying assay (Fig. 5C), which evaluates anxiety-like, repetitive, or exploratory behaviors, showed that both juvenile and adult *Shank3*^*Δex4–22*^ knockout mice buried significantly fewer marbles than wildtype and heterozygous mice (Fig. 5C, D, G). Moreover, reduced marble-burying behavior was observed in both sexes of *Shank3*^*Δex4–22*^ knockout mice (Fig. 5E, F, H), suggesting novelty-induced anxiety or reduced exploratory behavior, which aligns with similar behaviors observed in the open field (Figs. 1B-E and 2A-E) and elevated zero maze (Fig. 1K-N) tests. However, no significant difference was found in the number of marbles buried by wildtype and juvenile *Shank3*^*Δex4–22*^ knockout males (Fig. 5H).

### Spatial working memory is not disrupted by the absence of Shank3

Spatial working memory was evaluated through spontaneous exploration of the Y-maze. Although the total number of arm explorations was reduced in mice lacking Shank3 (Fig. 5I, J), there was no difference in the percentage of those explorations that were novel alternations (Fig. 5K, L). The reduction in the total number of arm entries observed in *Shank3*^*Δex4–22*^ knockout mice is consistent with reduced exploratory behavior observed in the open field (Fig. 2) and marble burying assay (Fig. 5C-H) for these same mice. However, the absence of any genotype effect on the percent of alternations (Fig. 5K-L), regardless of age or sex, suggests that spatial working memory is intact in the absence of Shank3.

### Mice lacking Shank3 display reduced social preference, but not social novelty

**Fig. 6:**
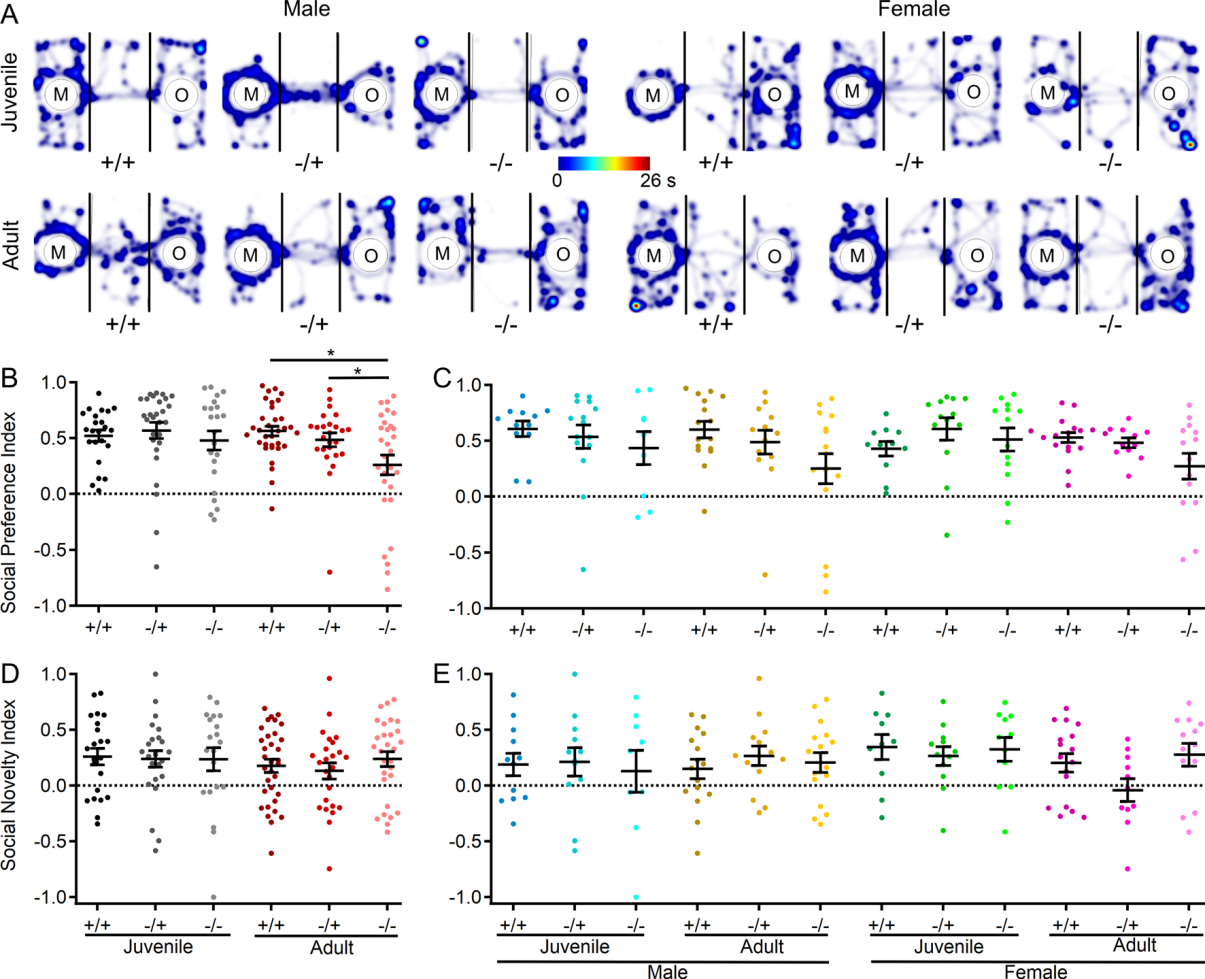
*Shank3*^*Δex4–22*^ KO mice develop reduced social preference with age in the three-chamber sociability assay. (**A**) Representative heatmaps of time spent in each area of the three-chamber arena for one mouse of each genotype, age, and sex. The color scale bar at the center applies to all heatmaps in the **A**. (**B-E**) Individual (circles) and mean ± SEM (black bars) of the social preference index (**B**,** C**) and social novelty index (**D**,** E**) for each genotype at both ages (**B**,** D**) and further separated by sex (**C**,** E**). *N* = 23–33 mice/group for each genotype at each age and *N* = 9–17 mice/group for each sex within each genotype at each age. *p < 0.05 for post-hoc test between genotypes with a Bonferroni correction

In a final assessment of behavior in *Shank3*^*Δex4–22*^ mice, a three-chamber sociability assay was conducted to evaluate both social preference (object vs. mouse) and social novelty preference (familiar mouse vs. novel mouse) [85]. In comparing the social preference index across groups, there was a general preference for social stimuli over non-social stimuli (Fig. 6A-C) and for novel over familiar mouse stimuli (Fig. 6D, E) across all three genotypes, both sexes and age groups. Although there were no significant main effects, there was a significant interaction between genotype and age on social preference behavior, with the social preference index for adult *Shank3*^*Δex4–22*^ knockout mice being significantly reduced relative to heterozygous and wildtype mice (Fig. 6B). In contrast, there were no genotype effects on preference for a novel target mouse over a familiar target mouse in the social novelty phase of the assay (Fig. 6D, E).

### Shank3 is present surrounding all cerebellar cortical mossy fiber terminals

**Fig. 7:**
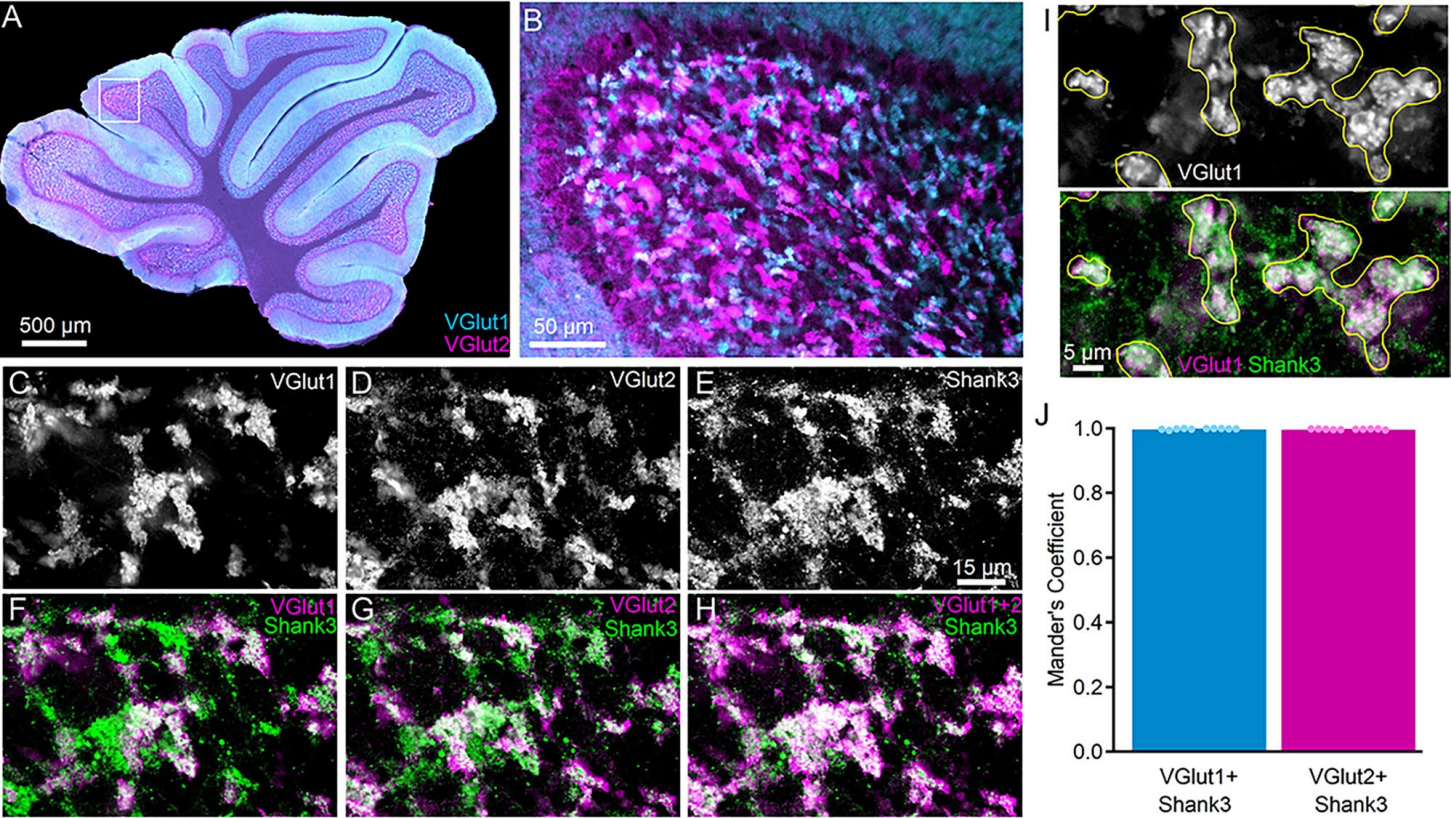
Shank3 is expressed at CGC dendrites around VGlut1- and VGlut2-positive mossy fibers (MF) terminals. (**A**,** B**) Confocal fluorescence images at 4x (**A**) and 20x (**B**) demonstrating expression of VGlut1 (cyan) and VGlut2 (magenta) throughout the internal granule cell layer at mossy fiber terminals. (**C-H**) Grayscale (**C-E**) and pseudocolor (**F-H**) 60x confocal fluorescence single-plane images of the same image location in the internal granule cell layer in parasagittal sections labeled with VGlut1, VGlut2, and Shank3. Fluorescence color is assigned to enhance contrast in comparing magenta and green. (**F-H**) Shank3 is expressed around VGlut1- and VGlut2-expressing terminals. (**I)** Example of how VGlut1-positive (white, top) terminals were used to define ROIs (yellow) for Shank3 colocalization analysis. (**J**) Individual (circles) and mean ± SEM (bars) Mander’s coefficient for each analyzed image reflect similar colocalization of Shank3 at VGlut1- and VGlut2-expressing mossy fibers. VGlut1-expressing (281 terminals) and VGlut2-expressing (285 terminals) were evaluated from *n* = 10 images per mossy fiber marker from *N* = 5 C57BL/6J mice

The *Shank3* gene is expressed in specific brain regions, including the striatum, cortex, hippocampus, thalamus, and cerebellum among others [13, 33]. However, despite the involvement of the cerebellum in multiple motor and non-motor processes, little is known about the distribution and function of Shank3 in the cerebellum other than its expression in CGCs [33, 44–46, 48]. To understand where Shank3 is expressed in CGCs, parasagittal sections of wildtype C57BL/6J mice were immunostained for Shank3 and markers of the two major classes of cerebellar cortical mossy fiber terminals (VGlut1 and VGlut2) that provide the primary input to CGCs (Fig. 7). Since VGlut1- and VGlut2-expressing MFs may arise from different sources [90], it was not surprising that their staining rarely overlapped within the internal granule cell layer (Fig. 7A, B). However, at nearly all MFs, regardless of whether they were of the VGlut1 or VGlut2 type, Shank3 was expressed surrounding each terminal type (Fig. 7C-H), indicating the broad presence of Shank3 at all inputs to CGCs. Quantitative colocalization analysis of Shank3 with either VGlut1 or VGlut2 supported this observation and that there was no significant difference (*t* (18) = 1.376, *p* = 0.186) in the colocalization of Shank3 with VGlut1 (Mander’s Coefficient = 0.9986 ± 0.0005; 281 ROIs from *n* = 10 images from *N* = 5 mice) or VGlut2 (Mander’s Coefficient = 0.9977 ± 0.0004, 285 ROIs from *n* = 10 images from *N* = 5 mice).

### Spontaneous excitatory synaptic events are larger in the absence of Shank3

**Fig. 8:**
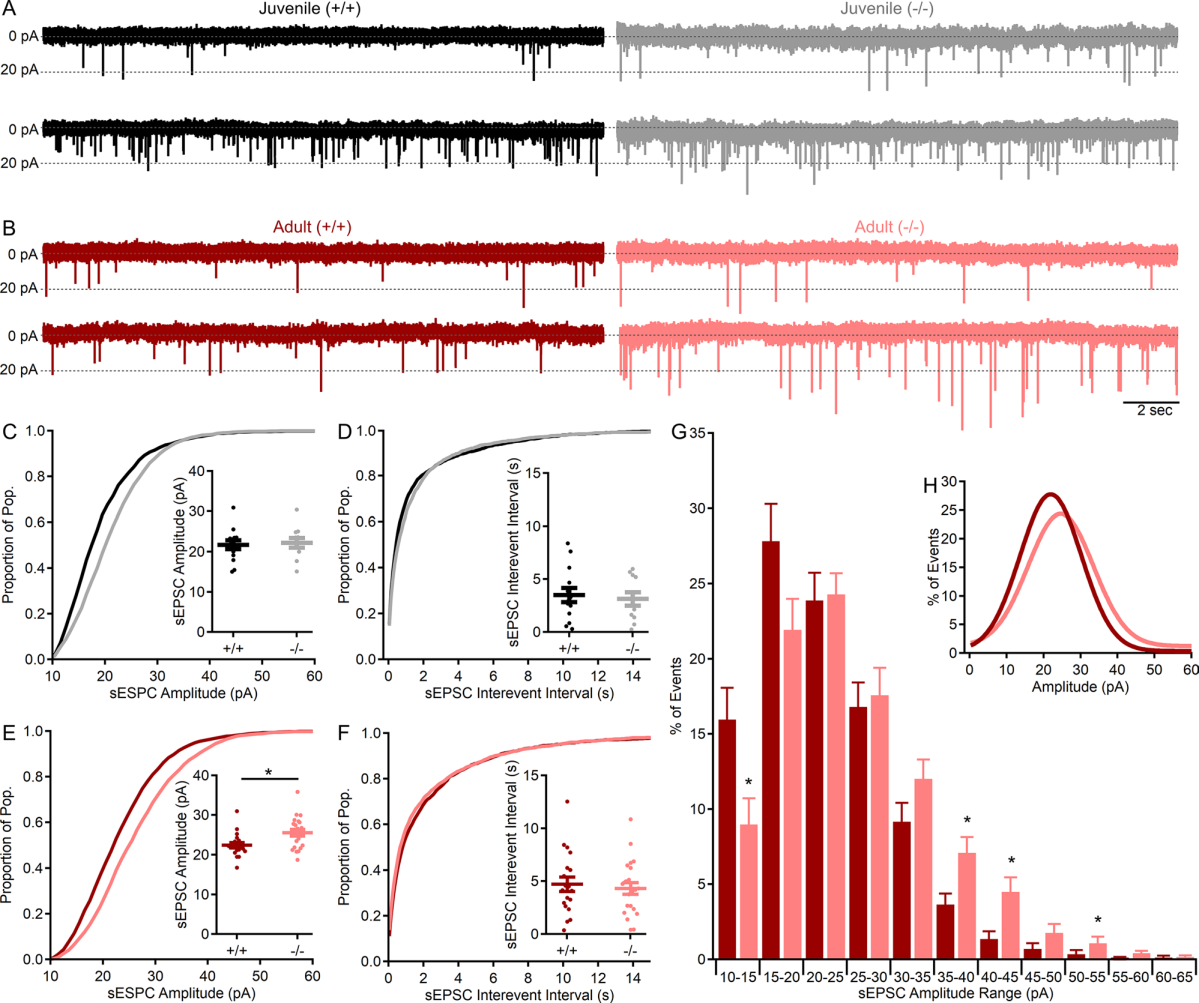
MF–CGC sEPSC amplitude is augmented in adult mice lacking Shank3. (**A**,** B**) Representative (20 s) traces of CGC sEPSCs (in 10µM gabazine) recorded from two genotypes each juvenile (**A**, black, gray) and adult (**B**, red, light red) wildtype (+/+) and knockout (-/-) *Shank3*^*Δex4–22*^ mice. Each trace is from a different CGC. (**C-F**) Cumulative distribution histograms for all events for each group with corresponding inset individual (circles) and mean ± SEM (bars) for sEPSC amplitudes (**C**,** E**) and interevent intervals (**D**,** F**) from juvenile (**C**,** D**) and adult (**E**,** F**) wildtype (+/+) and knockout (-/-) *Shank3*^*Δex4–22*^ mice. (**G**) Average normalized (to total event number) distribution histogram for sEPSC values from each CGC with the Gaussian fit of the averaged distribution provided in the inset (**H**). *n* = 19–22 cells/genotype from *N* = 11–14 adult mice and *n* = 11–14 cells/genotype from *N* = 6–9 juvenile mice. For comparison of mean group sEPSC values (**C-F** insets) or averaged sEPSC histogram bin percentages (**G**) *p < 0.05 for t-test between genotypes

With the expression of Shank3 in CGC dendrites encasing all glutamatergic MF terminals (Fig. 7), spontaneous non-NMDA receptor-mediated excitatory synaptic currents (sEPSCs) were evaluated in wildtype and *Shank3*^*Δex4–22*^ knockout mice at both ages to identify a relationship between behavioral phenotype and cerebellar glutamatergic CGC-MF function. Pharmacologically isolated sEPSCs (in the presence of 10 µM gabazine) recorded from CGCs (Fig. 8A; *n* = 1730 events in 14 cells from *N* = 9 wildtype mice and *n* = 1845 events in 11 cells from *N* = 6 knockout) of juvenile mice were comparable in both amplitude (Fig. 8C; *t*(23) = -0.29, *p* = 0.77) and frequency (Fig. 8D; t(23) = 0.39, *p* = 0.70). However, sEPSCs from adult CGCs (Fig. 8B; *n* = 2122 events in 19 cells from *N* = 11 wildtype mice and *n* = 2694 events in 22 cells from 14 knockout mice) were significantly larger in *Shank3*^*Δex4–22*^ knockout mice (Fig. 8E; *t*(39) = -2.82, *p* = 0.008), but occurred at similar frequencies in both genotypes (Fig. 8F; *t*(39) = 0.84, *p* = 0.40). The increase in the mean sEPSC amplitude averaged per cell (Fig. 8E inset) can also be observed in the rightward shift in the cumulative amplitude distribution histogram for all sEPSC amplitudes (Fig. 8E), with a similar trend observed in the amplitude distribution from juvenile animals (Fig. 8C).

Because the sEPSCs may be action potential-dependent or -independent and, as a result, single or multi-quantal, the distribution of sEPSC amplitudes was evaluated further (Fig. 8G, H). To reduce the impact of individual cells with high or low numbers of sEPSC events, an individual event amplitude histogram (5 pA bin) was constructed for each CGC and normalized to that neuron’s total number of events, with the resulting normalized histogram averaged across groups (Fig. 8G, H). The averaged histograms have a single peak (Fig. 8H) which was shifted from 15 to 20 pA in CGCs from wildtype mice to 20–25 pA in adult knockout mice. Since sEPSCs in granule cells are in response to the release of single quanta [91], this single peak distribution of event amplitudes suggests that there is likely a specific postsynaptic augmentation of ~ 5 pA or 25%. This progressive augmentation of non-NMDA receptor-mediated sEPSC amplitude in adult mice with absent Shank3 suggests that there may be changes in the type or level of postsynaptic AMPA or kainate receptors at the CGC-MF synapse. The absence of any sEPSC frequency changes and the single peak amplitude distribution suggests that presynaptic release is unlikely to be affected, which is in line with a lack of Shank3 expression in MF terminals.

## Discussion

To understand how specific behaviors develop or decline throughout early adulthood in PMS and some forms of ASD, we evaluated the behavior of *Shank3*^*Δex4–22*^ mice [47], a pre-clinical animal model of PMS and ASD, at two specific time points (5–6 weeks and 3–5 months). Evaluation of wildtype, heterozygous, and knockout mice on behavioral assays across a range of domains, including anxiety-like, motor, exploratory, memory, and social behavior, revealed that behavioral changes in the absence of Shank3 fell into three categories. First, exploratory behavior (Figs. 2A-E and 5C-J), gait differences (Fig. 4), and repetitive behavior (Fig. 5A-H) were relatively well-established in juvenile mice, and little change was observed in these areas in adult cohorts. In contrast, loss of Shank3 led to increased anxiety-like behavior (Fig. 1A-E & J-N), disruption of motor coordination (Fig. 3), and reduced social preference (Fig. 6) that became more pronounced in adult mice relative to juvenile mice. Finally, Shank3 loss did not seem to impact freezing (Fig. 1F, G), fecal boli deposits (Fig. 1H, I), gross motor ability (e.g. locomotion speed; Fig. 2F, G), or short-term spatial memory (Fig. 5K, L).

With this broad assessment of how behavioral phenotype develops or declines in the absence of Shank3, we also sought to identify a neural mechanism that may contribute to or at least parallel this behavioral decline with age. Based on the relatively late-stage development of the cerebellum [92] and the cerebellum’s interaction with multiple other brain areas, we hypothesized that the absence of Shank3 from this circuit may be particularly consequential. Immunohistochemical assessment of Shank3 distribution revealed that it is present in CGC dendrites that encase nearly all glutamatergic MF inputs into the granule cell layer (Fig. 7) – a key site of signal integration in the cerebellum. Electrophysiological interrogation of this synapse revealed not only an enhanced MF-CGC synaptic response (Fig. 8), but also that this augmentation was specific to adult *Shank3*^*Δex4–22*^ mice, paralleling the exacerbation of changes in behaviors known to be modulated by the cerebellum: anxiety-like behavior, disrupted motor, and social behaviors. The sEPSCs analyzed here to evaluate MF-CGC functional changes in the absence of Shank3 may represent pre- and postsynaptic effects in principle. However, given that the expression of Shank3 is limited to CGCs at this synapse and that the sEPSCs are comparable to mEPSCs at MF-CGC synapses [91], we predict that the synaptic augmentation is likely due to the expression, trafficking, or subunit composition of non-NMDA receptors at the MF-CGC synapse.

Our behavioral findings in the adult group are largely in line with previous studies conducted on *Shank3*^*Δex4–22*^ mice at similar ages [46, 47, 93], particularly regarding open field locomotion, rotarod, beam balance, and gait. However, the magnitude of some genotype-dependent differences in behavior for adult mice was somewhat smaller in this study than in previous reports, which may be due to some methodological differences. First, the background strain of the mice used here was a mixed C57BL/6NJ as provided by the vendor, while others used *Shank3*^*Δex4–22*^ mice on a C57BL/6Tac [47] or C57BL/6J [46] background. Perhaps more importantly, the behavioral data collected in this study occurred during the active dark cycle and in low light conditions, rather than during the inactive light cycle as done by others [46, 47, 93] that may introduce additional circadian-related variables. The current study was also sufficiently powered to detect sex effects, which allowed for the identification of significant genotype-age-sex interactions that identified increased stereotyped grooming and reduced social preference behavior in *Shank3*^*Δex4–22*^ knockout mice.

Behavioral analysis of various *Shank3* mutant mouse lines, each lacking specific exons, has typically been conducted within single age cohorts, either in neonatal or adult mice. These findings have been summarized elsewhere, and the role of isoforms is discussed in detail below [47]. However, recently some groups have specifically compared the behavioral phenotypes of the *Shank3*^*Δex11*^ [26, 27], *Shank3*^*Δex21*^ [28], and *Shank3*^*Δex4–22*^ [29] lines across age groups (Table 1). Although specific comparisons between ages were not made, the extensive dataset from the *Shank3*^*Δex4–22*^ originating reference Drapeau et al., 2018 [47] is included in Table 1 for comparison and due to behavioral testing over a broad age range. While the methodological details of each of these studies differ from one another with respect to age ranges, all were performed during the inactive light phase or did not clarify the timing, and some retested the same cohort of mice. Three of these multi-timepoint studies [26–28] evaluated behavior in mouse lines that only lack some Shank3 isoforms, which may explain why only a limited subset of behaviors are evaluated and/or found to be different across genotypes regardless of age. Unlike prior longitudinal studies [26–29], where the same animals were subjected to the same behavioral experiments at different ages, potentially influencing the behavioral results at older ages due to task familiarization and learning ability, our findings based on separate age groups at two different time points provide a novel and more robust characterization of the behavioral changes between juvenile and adult knockout mice. Since two other studies evaluated a motor, social, exploratory, and anxiety-like behavior in the *Shank3*^*Δex4–22*^ mouse at earlier developmental time points (2–8 weeks [29]) or once at variable time points (12–35 weeks [47]), the current study may combine with this work, to provide a more comprehensive understanding of how behavioral domains are affected by the loss of all Shank3 isoforms over time.

**Table 1:** Comparison of studies demonstrating behavioral changes in *Shank3* mutant mouse models with age

Publication		Multi-Time Point Comparison Studies				Current Study	Originating Study
		Ferhat et al., 2023	Bauer et al., 2023	Thabault et al., 2023	Contestabile et al., 2023	Kshetri et al.,	Drapeau et al., 2018
*Shank3* Deletion		Δ11	Δ11	Δ21	Δ4–22	Δ4–22	Δ4–22
Isoforms Deleted		a, b,c	a, b,c	a, c,d, e,f	a, b,c, d,e, f	a, b,c, d,e, f	a, b,c, d,e, f
Testing Light Cycle		Light	N/A	N/A	Light	Dark	Light
Ages Compared (weeks)		12, 32, 52	4–9, 13–18	10, 20,40	2–8	5–7, 12–20	3, 12–35
Separate Age Cohorts		Y/N	N	Y	N/A	Y	N
Sexes		M/F	M/F	M/F	M/F	M/F	M/F
Motor Function	Rotarod		↓		↓	↓	↓
	Beam Balance					↓	↓
	Gait					−	−
	Speed					−	−
	Strength		↓				↓
Social	3 Chamber/Free Social Preference	−	−		↓	↓	−
	3 Chamber Social Novelty					−	−
	Sociosexual	↑					↓
	Ultrasonic Vocalizations	−	−				−
Exploratory	Locomotion	↓	↓		−	↓	↓
	Marble Burying		↓			↓	↓
Anxiety-like	Open Field Center Time		↓		↓	↓	↓
	Zero/Plus Maze	−			↓	↓	↓
Repetitive/ Stereotyped	Grooming	↑	↑	↑		↑	↑
	Rearing			↑			
	Nestlet Shredding		↑				↓
Memory	Y-Maze	−				−	−
	Barnes/Star Maze	−	−				↓

*Abbreviations* Y, Yes; N, No; N/A, Not available; M, Male; F, Female; ↑, increase; ↓, decrease; –, no change in tested behaviors due to reduced Shank3 expression

Since each Shank3 isoform is preferentially expressed at different levels across discrete brain areas [33], the behavioral impact of deleting a subset of exons and related isoforms depends on the role each isoform plays in different brain areas at specific times. For example, *Shank3*-mutant mice missing exons 4–9 that only lack isoforms A-B (ankyrin-containing) [35, 36, 42–45] more commonly display stereotyped grooming behavior and altered social interactions. In contrast, mice with mutations affecting exons 11–22 that lack expression of isoforms C-D (non-ankyrin containing) [34, 46, 47, 94, 95] more often display heightened avoidance behaviors, anxiety, deficits in sensory processing, and poor performance on cerebellar-dependent motor tasks [46, 47]. Variation in isoform expression over time is also a key consideration in addition to the location of isoform expression. With the need to address all isoforms and account for key temporal progressions of isoform expression, the present study fills an important gap in the current literature.

With the range of behavioral domains affected in mice with germline *Shank3* exon deletions, the brain regions most disrupted by the loss of Shank3 to affect these other behaviors remain to be identified. The integration of timelines for behavioral phenotype development and isoform expression along with knowledge of brain regional variation in isoform expression perhaps guide future work aimed at addressing this issue. The behavioral regression observed in both humans [20–25] and mice (Table 1, this study) occurs over a time when isoform C and D expression is increasing to its steady state peak during adolescence and early adulthood [33]. This timeframe also aligns with the final stages in the development of the cerebellar cortex, where Shank3 is expressed exclusively in cerebellar granule cells as isoforms C and D [44, 65, 96–98]. Guided by these data, our findings not only demonstrate the ubiquitous presence of Shank3 at all MF-CGC synapses (Fig. 7), key sites for sensorimotor integration within the cerebellar circuit but also reveal Shank3-dependent changes in the function of this synapse (Fig. 8) that align with the timing of behavioral phenotype regression across juvenile and early adulthood. This novel finding of disrupted glutamatergic synaptic function in the absence of Shank3 provides evidence for cerebellar disruption in this animal model of PMS and ASD, while also highlighting the need to determine the specific cause of this synaptic augmentation (subunit change, receptor density increase, etc.).

Determining the Shank3-brain region interactions that drive Shank3-dependent changes in anxiety, motor function, social interactions, communication, and some forms of learning is crucial for understanding and treating PMS, and possibly ASD. The temporal relationship between cerebellar synaptic functional changes and behavioral regression in *Shank3*^*Δex4–22*^ mice suggests cerebellar involvement. Recent studies [21, 23] have reported a significant correlation between age and the prevalence and severity of regression in cases of PMS. Interestingly, regression was primarily observed to impact fine and gross motor function and language skills, which are largely considered to be under cerebellar control. Although these symptoms typically begin at a young age, they tend to worsen during adolescence. Multiple studies indicate that the pathophysiology of the cerebellum, likely via these non-motor connections, may be involved in the etiology of ASD [10, 57–64]. Changes in cerebellar cortical size [99, 100], development [101–103], and Purkinje cell (PC, principal cortical efferent) density [104] have all been linked to ASD diagnoses. In addition, cerebellar damage at birth [57, 105] and dysfunction in known cerebellar-dependent behaviors or differences in cerebellar activity (e.g. fMRI) are also linked to ASD [106–112].

### Limitations

Despite the significant findings of this study, other behavioral assessments, including evaluations of muscle strength (e.g. grip strength), cognitive function (e.g. Barnes or Morris Water maze), and communication (e.g. ultrasonic vocalizations) were not conducted. These tests could provide comprehensive insights into even broader phenotypic manifestations associated with the loss of Shank3 at different developmental time points and in both sexes. Future investigations incorporating these behavioral tests are essential to delineate the multifaceted impacts of Shank3 on overall behavioral functions. Furthermore, the role of Shank3 in the cerebellum remains under-explored. While this study focused on Shank3’s influence on synaptic mechanisms at MF-CGC synapses, the specific pathways and cellular mechanisms through which Shank3 modulates synaptic function in this context remain to be fully elucidated. Further research exploring structural and functional changes at MF-CGC synapses is necessary to uncover the precise modulatory effects of Shank3. While these data point to changes in CGC synaptic function and behavioral phenotype that appear to develop in parallel along similar timeframes, the degree to which the cerebellar-specific dysfunction may cause these behavioral differences in the absence of Shank3 has not yet been assessed.

## Conclusions

The behavioral data presented here provide a comprehensive view of behavioral differences based on Shank3 expression across multiple domains and in both sexes in mice. The assessment of these behaviors at juvenile and adult time points reveals domain-specific behavioral regression that aligns with analogous time points in humans. Together, these data offer a comprehensive understanding of Shank3-dependent behavioral changes and their variation over time when assessed during an animal’s active (dark) phase. Finally, to identify functional changes corresponding to genotypic and developmental behavioral phenotypes, electrophysiology data identify an augmentation of the MF-CGC synapse that may impact cerebellar input integration and downstream modulation of multiple motor and non-motor circuits/processes.

## Electronic Supplementary Material

Below is the link to the electronic supplementary material.

Supplementary Material 1: Statistical results for all behavioral analyses.

## Data Availability

The datasets used and/or analyzed during the current study are available from the corresponding author on reasonable request with statistical analysis results included with this published article’s supplementary information files.

## References

[1] Maenner MJ, Shaw KA, Baio J, Washington A, Patrick M, DiRienzo M, et al. Prevalence of autism spectrum disorder among children aged 8 years-autism and developmental disabilities monitoring network, 11 sites, United States, 2016. MMWR Surveillance Summaries. 2020;69:1–12. 10.15585/mmwr.ss6904a1 PMC7119644 32214087

[2] Varghese M, Keshav N, Jacot-Descombes S, Warda T, Wicinski B, Dickstein DL, et al. Autism spectrum disorder: neuropathology and animal models. Acta Neuropathol. 2017;134:537–66. 28584888 10.1007/s00401-017-1736-4 PMC5693718

[3] Pinto D, Delaby E, Merico D, Barbosa M, Merikangas A, Klei L, et al. Convergence of genes and cellular pathways dysregulated in autism spectrum disorders. Am J Hum Genet. 2014;94:677–94. 24768552 10.1016/j.ajhg.2014.03.018 PMC4067558

[4] De Rubeis S, Buxbaum JD. Genetics and genomics of autism spectrum disorder: embracing complexity. Hum Mol Genet. 2015;24:R24–31. 26188008 10.1093/hmg/ddv273 PMC4675826

[5] Won H, Mah W, Kim E. Autism spectrum disorder causes, mechanisms, and treatments: focus on neuronal synapses. Front Mol Neurosci. 2013;6:19. 23935565 10.3389/fnmol.2013.00019 PMC3733014

[6] Larsen E, Menashe I, Ziats MN, Pereanu W, Packer A, Banerjee-Basu S. A systematic variant annotation approach for ranking genes associated with autism spectrum disorders. Mol Autism. 2016;7:44. 27790361 10.1186/s13229-016-0103-y PMC5075177

[7] Satterstrom FK, Kosmicki JA, Wang J, Breen MS, De Rubeis S, An J-Y, et al. Large-Scale Exome Sequencing Study Implicates Both Developmental and Functional Changes in the Neurobiology of Autism. Cell. 2020;180:568–e58423. 31981491 10.1016/j.cell.2019.12.036 PMC7250485

[8] Feliciano P, Zhou X, Astrovskaya I, Turner TN, Wang T, Brueggeman L et al. Exome sequencing of 457 autism families recruited online provides evidence for autism risk genes. NPJ Genom Med. 2019;4. 10.1038/s41525-019-0093-8 PMC6707204 31452935

[9] Betancur C, Buxbaum JD. SHANK3 haploinsufficiency: a common but underdiagnosed highly penetrant monogenic cause of autism spectrum disorders. Mol Autism. 2013;4:17. 23758743 10.1186/2040-2392-4-17 PMC3695795

[10] Hulbert SW, Jiang Y. Cellular and Circuitry Bases of Autism: Lessons Learned from the Temporospatial Manipulation of Autism Genes in the Brain. Neurosci Bull. 2017;33:205–18. 28271437 10.1007/s12264-017-0112-7 PMC5360850

[11] Leblond CS, Nava C, Polge A, Gauthier J, Huguet G, Lumbroso S, et al. Meta-analysis of SHANK Mutations in Autism Spectrum Disorders: a gradient of severity in cognitive impairments. PLoS Genet. 2014;10:e1004580. 25188300 10.1371/journal.pgen.1004580 PMC4154644

[12] Durand CM, Betancur C, Boeckers TM, Bockmann J, Chaste P, Fauchereau F, et al. Mutations in the gene encoding the synaptic scaffolding protein SHANK3 are associated with autism spectrum disorders. Nat Genet. 2007;39:25–7. 17173049 10.1038/ng1933 PMC2082049

[13] Monteiro P, Feng G. SHANK proteins: roles at the synapse and in autism spectrum disorder. Nat Rev Neurosci. 2017;18:147–57. 28179641 10.1038/nrn.2016.183

[14] Kolevzon A, Angarita B, Bush L, Wang AT, Frank Y, Yang A, et al. Phelan- McDermid syndrome: a review of the literature and practice parameters for medical assessment and monitoring. J Neurodev Disord. 2014;6:39. 10.1186/1866-1955-6-39 PMC4362650 25784960

[15] Phelan MC, Thomas GR, Saul RA, Rogers RC, Taylor HA, Wenger DA, et al. Cytogenetic, biochemical, and molecular analyses of a 22q13 deletion. Am J Med Genet. 1992;43:872–6. 1353666 10.1002/ajmg.1320430524

[16] Phelan K, McDermid HE. The 22q13.3 Deletion Syndrome (Phelan-McDermid Syndrome). Mol Syndromol. 2012;2:186–201. 22670140 10.1159/000334260 PMC3366702

[17] Costales JL, Kolevzon A. Phelan–McDermid Syndrome and SHANK3: Implications for Treatment. Neurotherapeutics. 2015;12:620–30. 25894671 10.1007/s13311-015-0352-z PMC4489957

[18] Zwanenburg RJ, Van Den Ruiter SAJ, Flapper BCT, Van Ravenswaaij-Arts CMA. Developmental phenotype in Phelan- McDermid (22q13.3 deletion) syndrome: A systematic and prospective study in 34 children. J Neurodev Disord. 2016;8:1–12. 27118998 10.1186/s11689-016-9150-0 PMC4845478

[19] Soorya L, Kolevzon A, Zweifach J, Lim T, Dobry Y, Schwartz L, et al. Prospective investigation of autism and genotype-phenotype correlations in 22q13 deletion syndrome and SHANK3 deficiency. Mol Autism. 2013;4:1–17. 23758760 10.1186/2040-2392-4-18 PMC3707861

[20] Kolevzon A, Delaby E, Berry-Kravis E, Buxbaum JD, Betancur C. Neuropsychiatric decompensation in adolescents and adults with Phelan-McDermid syndrome: a systematic review of the literature. Mol Autism. 2019;10:50. 31879555 10.1186/s13229-019-0291-3 PMC6930682

[21] Dille Y, Lagae L, Swillen A, Buggenhout G, Van. Neurodevelopmental profile and stages of regression in Phelan-McDermid syndrome. Dev Med Child Neurol. 2023;65:917–25. 36477723 10.1111/dmcn.15482

[22] Kohlenberg TM, Trelles MP, McLarney B, Betancur C, Thurm A, Kolevzon A. Psychiatric illness and regression in individuals with Phelan-McDermid syndrome. J Neurodev Disord. 2020;12:7. 32050889 10.1186/s11689-020-9309-6 PMC7014655

[23] Landlust AM, Koza SA, Carbin M, Walinga M, Robert S, Cooke J, et al. Parental perspectives on Phelan-McDermid syndrome: Results of a worldwide survey. Eur J Med Genet. 2023;66:104771. 37120079 10.1016/j.ejmg.2023.104771

[24] De Rubeis S, Siper PM, Durkin A, Weissman J, Muratet F, Halpern D, et al. Delineation of the genetic and clinical spectrum of Phelan-McDermid syndrome caused by SHANK3 point mutations. Mol Autism. 2018;9:31. 29719671 10.1186/s13229-018-0205-9 PMC5921983

[25] Reierson G, Bernstein J, Froehlich-Santino W, Urban A, Purmann C, Berquist S, et al. Characterizing regression in Phelan McDermid Syndrome (22q13 deletion syndrome). J Psychiatr Res. 2017;91:139–44. 28346892 10.1016/j.jpsychires.2017.03.010 PMC5469716

[26] Ferhat A-T, Verpy E, Biton A, Forget B, De Chaumont F, Mueller F, et al. Excessive self-grooming, gene dysregulation and imbalance between the striosome and matrix compartments in the striatum of Shank3 mutant mice. Front Mol Neurosci. 2023;16:1–17. 10.3389/fnmol.2023.1139118 PMC10061084 37008785

[27] Bauer HF, Delling JP, Bockmann J, Boeckers TM, Schön M. Development of sex- and genotype-specific behavioral phenotypes in a Shank3 mouse model for neurodevelopmental disorders. Front Behav Neurosci. 2023;16:1–14. 10.3389/fnbeh.2022.1051175 PMC9868822 36699652

[28] Thabault M, Turpin V, Balado É, Fernandes-Gomes C, Huot A-L, Cantereau A, et al. Age-related behavioural and striatal dysfunctions in Shank3∆C/∆C mouse model of autism spectrum disorder. Eur J Neurosci. 2023;57:607–18. 36656446 10.1111/ejn.15919

[29] Contestabile A, Casarotto G, Musardo S, Espinosa P, Maltese F, Jiang Yhui, et al. Shank3 deficits in the anteromedial bed nucleus of the stria terminalis trigger an anxiety phenotype in mice. Eur J Neurosci. 2023;57:1966–79. 37165567 10.1111/ejn.16043

[30] Lim S, Naisbitt S, Yoon J, Hwang JI, Suh PG, Sheng M, et al. Characterization of the Shank family of synaptic proteins. Multiple genes, alternative splicing, and differential expression in brain and development. J Biol Chem. 1999;274:29510–8. 10506216 10.1074/jbc.274.41.29510

[31] Naisbitt S, Kim E, Tu JC, Xiao B, Sala C, Valtschanoff J, et al. Shank, a novel family of postsynaptic density proteins that binds to the NMDA receptor/PSD-95/GKAP complex and cortactin. Neuron. 1999;23:569–82. 10433268 10.1016/s0896-6273(00)80809-0

[32] Grabrucker AM, Schmeisser MJ, Schoen M, Boeckers TM. Postsynaptic ProSAP/Shank scaffolds in the cross-hair of synaptopathies. Trends Cell Biol. 2011;21:594–603. 21840719 10.1016/j.tcb.2011.07.003

[33] Wang X, Xu Q, Bey AL, Lee Y, Jiang YH. Transcriptional and functional complexity of Shank3 provides a molecular framework to understand the phenotypic heterogeneity of SHANK3 causing autism and Shank3 mutant mice. Mol Autism. 2014;5:1–14. 25071925 10.1186/2040-2392-5-30 PMC4113141

[34] Mei Y, Monteiro P, Zhou Y, Kim J-A, Gao X, Fu Z, et al. Adult restoration of Shank3 expression rescues selective autistic-like phenotypes. Nature. 2016;530:481–4. 26886798 10.1038/nature16971 PMC4898763

[35] Jaramillo TC, Speed HE, Xuan Z, Reimers JM, Liu S, Powell CM. Altered Striatal Synaptic Function and Abnormal Behaviour in Shank3 Exon4-9 Deletion Mouse Model of Autism. Autism Res. 2016;9:350–75. 26559786 10.1002/aur.1529 PMC4857590

[36] Drapeau E, Dorr NP, Elder GA, Buxbaum JD. Absence of strong strain effects in behavioral analyses of Shank3-deficient mice. Dis Model Mech. 2014;7:667–81. 24652766 10.1242/dmm.013821 PMC4036474

[37] Duffney LJ, Zhong P, Wei J, Matas E, Cheng J, Qin L, et al. Autism-like Deficits in Shank3-Deficient Mice Are Rescued by Targeting Actin Regulators. Cell Rep. 2015;11:1400–13. 26027926 10.1016/j.celrep.2015.04.064 PMC4464902

[38] Vicidomini C, Ponzoni L, Lim D, Schmeisser MJ, Reim D, Morello N, et al. Pharmacological enhancement of mGlu5 receptors rescues behavioral deficits in SHANK3 knock-out mice. Mol Psychiatry. 2017;22:689–702. 27021819 10.1038/mp.2016.30 PMC5014121

[39] Jaramillo TC, Speed HE, Xuan Z, Reimers JM, Escamilla CO, Weaver TP, et al. Novel Shank3 mutant exhibits behaviors with face validity for autism and altered striatal and hippocampal function. Autism Res. 2017;10:42–65. 27492494 10.1002/aur.1664 PMC5274551

[40] Kloth AD, Badura A, Li A, Cherskov A, Connolly SG, Giovannucci A, et al. Cerebellar associative sensory learning defects in five mouse autism models. Elife. 2015;4:e06085. 26158416 10.7554/eLife.06085 PMC4512177

[41] Bey AL, Wang X, Yan H, Kim N, Passman RL, Yang Y, et al. Brain region-specific disruption of Shank3 in mice reveals a dissociation for cortical and striatal circuits in autism-related behaviors. Transl Psychiatry. 2018;8:94. 29700290 10.1038/s41398-018-0142-6 PMC5919902

[42] Bozdagi O, Sakurai T, Papapetrou D, Wang X, Dickstein DL, Takahashi N, et al. Haploinsufficiency of the autism-associated Shank3 gene leads to deficits in synaptic function, social interaction, and social communication. Mol Autism. 2010;1:15. 21167025 10.1186/2040-2392-1-15 PMC3019144

[43] Yang M, Bozdagi O, Scattoni ML, Wöhr M, Roullet FI, Katz AM, et al. Reduced excitatory neurotransmission and mild autism-relevant phenotypes in adolescent Shank3 null mutant mice. J Neurosci. 2012;32:6525–41. 22573675 10.1523/JNEUROSCI.6107-11.2012 PMC3362928

[44] Peça J, Feliciano C, Ting JT, Wang W, Wells MF, Venkatraman TN, et al. Shank3 mutant mice display autistic-like behaviours and striatal dysfunction. Nature. 2011;472:437–42. 21423165 10.1038/nature09965 PMC3090611

[45] Lee J, Chung C, Ha S, Lee D, Kim D-Y, Kim H, et al. Shank3-mutant mice lacking exon 9 show altered excitation/inhibition balance, enhanced rearing, and spatial memory deficit. Front Cell Neurosci. 2015;9:94. 25852484 10.3389/fncel.2015.00094 PMC4365696

[46] Wang X, Bey AL, Katz BM, Badea A, Kim N, David LK, et al. Altered mGluR5-Homer scaffolds and corticostriatal connectivity in a Shank3 complete knockout model of autism. Nat Commun. 2016;7:11459. 27161151 10.1038/ncomms11459 PMC4866051

[47] Drapeau E, Riad M, Kajiwara Y, Buxbaum JD. Behavioral Phenotyping of an Improved Mouse Model of Phelan-McDermid Syndrome with a Complete Deletion of the Shank3 Gene. eNeuro. 2018;5. 10.1523/ENEURO.0046-18.2018 PMC6175061 30302388

[48] Böckers TM, Mameza MG, Kreutz MR, Bockmann J, Weise C, Buck F, et al. Synaptic scaffolding proteins in rat brain: Ankyrin repeats of the multidomain Shank protein family interact with the cytoskeletal protein α-fodrin. J Biol Chem. 2001;276:40104–12. 11509555 10.1074/jbc.M102454200

[49] Burgoyne RD, Graham ME, Cambray-Deakin M. Neurotrophic effects of NMDA receptor activation on developing cerebellar granule cells. J Neurocytol. 1993;22:689–95. 7903688 10.1007/BF01181314

[50] Komuro H, Rakic P. Modulation of neuronal migration by NMDA receptors. Science. 1993;260:95–7. 8096653 10.1126/science.8096653

[51] D’Angelo E, De Zeeuw CI. Timing and plasticity in the cerebellum: focus on the granular layer. Trends Neurosci. 2009;32:30–40. 18977038 10.1016/j.tins.2008.09.007

[52] Lisman J. Long-term potentiation: Outstanding questions and attempted synthesis. Philosophical Trans Royal Soc B: Biol Sci. 2003;358:829–42. 10.1098/rstb.2002.1242 PMC1693147 12740130

[53] D’Errico A, Prestori F, D’Angelo E. Differential induction of bidirectional long-term changes in neurotransmitter release by frequency-coded patterns at the cerebellar input. J Physiol. 2009;587:5843–57. 19858226 10.1113/jphysiol.2009.177162 PMC2808544

[54] Rossi P, Sola E, Taglietti V, Borchardt T, Steigerwald F, Utvik JK, et al. NMDA receptor 2 (NR2) C-terminal control of NR open probability regulates synaptic transmission and plasticity at a cerebellar synapse. J Neurosci. 2002;22:9687–97. 12427824 10.1523/JNEUROSCI.22-22-09687.2002 PMC6757821

[55] Rossi P, D’Angelo E, Taglietti V. Differential Long-lasting Potentiation of the NMDA and Non-NMDA Synaptic Currents Induced by Metabotropic and NMDA Receptor Coactivation in Cerebellar Granule Cells. Eur J Neurosci. 1996;8:1182–9. 8752588 10.1111/j.1460-9568.1996.tb01286.x

[56] Kita K, Albergaria C, Machado AS, Carey MR, Müller M, Delvendahl I. Glua4 facilitates cerebellar expansion coding and enables associative memory formation. Elife. 2021;10. 10.7554/eLife.65152 PMC8291978 34219651

[57] Wang SS-H, Kloth AD, Badura A. The cerebellum, sensitive periods, and autism. Neuron. 2014;83:518–32. 25102558 10.1016/j.neuron.2014.07.016 PMC4135479

[58] Mosconi MW, Wang Z, Schmitt LM, Tsai P, Sweeney J. a. The role of cerebellar circuitry alterations in the pathophysiology of autism spectrum disorders. Front Neurosci. 2015;9:296. 10.3389/fnins.2015.00296 PMC4555040 26388713

[59] Hampson DR, Blatt GJ. Autism spectrum disorders and neuropathology of the cerebellum. Front Neurosci. 2015;9:420. 26594141 10.3389/fnins.2015.00420 PMC4635214

[60] Becker EBE, Stoodley CJ. Autism spectrum disorder and the cerebellum. Int Rev Neurobiol. 2013;113:1–34. 1st ed. 24290381 10.1016/B978-0-12-418700-9.00001-0

[61] Tsai PT. Autism and cerebellar dysfunction: Evidence from animal models. Semin Fetal Neonatal Med. 2016;21:349–55. 27179922 10.1016/j.siny.2016.04.009

[62] D’Mello AM, Stoodley CJ. Cerebro-cerebellar circuits in autism spectrum disorder. Front Neurosci. 2015;9:408. 26594140 10.3389/fnins.2015.00408 PMC4633503

[63] Fatemi SH, Aldinger KA, Ashwood P, Bauman ML, Blaha CD, Blatt GJ, et al. Consensus paper: pathological role of the cerebellum in autism. Cerebellum. 2012;11:777–807. 22370873 10.1007/s12311-012-0355-9 PMC3677555

[64] Hannant P, Tavassoli T, Cassidy S. The Role of Sensorimotor Difficulties in Autism Spectrum Conditions. Front Neurol. 2016;7:124. 27559329 10.3389/fneur.2016.00124 PMC4978940

[65] Menashe I, Grange P, Larsen EC, Banerjee-Basu S, Mitra PP. Co-expression profiling of autism genes in the mouse brain. PLoS Comput Biol. 2013;9:e1003128. 23935468 10.1371/journal.pcbi.1003128 PMC3723491

[66] De Zeeuw CI, Hoebeek FE, Bosman LWJ, Schonewille M, Witter L, Koekkoek SK. Spatiotemporal firing patterns in the cerebellum. Nat Rev Neurosci. 2011;12:327–44. 21544091 10.1038/nrn3011

[67] Apps R, Hawkes R. Cerebellar cortical organization: a one-map hypothesis. Nat Rev Neurosci. 2009;10:670–81. 19693030 10.1038/nrn2698

[68] Pisano TJ, Dhanerawala ZM, Kislin M, Bakshinskaya D, Engel EA, Lee J et al. Homologous organization of cerebellar pathways to sensory, motor, and associative forebrain. Cell Rep. 2021;36(12):109721. 10.1016/j.celrep.2021.109721 PMC8506234 34551311

[69] Kang S, Jun S, Baek SJ, Park H, Yamamoto Y, Tanaka-Yamamoto K. Recent Advances in the Understanding of Specific Efferent Pathways Emerging From the Cerebellum. Front Neuroanat. 2021;15. 10.3389/fnana.2021.759948 PMC8716603 34975418

[70] Fujita H, Kodama T, Du Lac S. Modular output circuits of the fastigial nucleus for diverse motor and nonmotor functions of the cerebellar vermis. Elife. 2020;9:1–91. 10.7554/eLife.58613 PMC7438114 32639229

[71] Judd EN, Lewis SM, Person AL. Diverse inhibitory projections from the cerebellar interposed nucleus. Elife. 2021;10. 10.7554/eLife.66231 PMC8483738 34542410

[72] Strick PL, Dum RP, Fiez JA. Cerebellum and nonmotor function. Annu Rev Neurosci. 2009;32:413–34. 19555291 10.1146/annurev.neuro.31.060407.125606

[73] Caligiore D, Pezzulo G, Baldassarre G, Bostan AC, Strick PL, Doya K, et al. Consensus Paper: Towards a Systems-Level View of Cerebellar Function: the Interplay Between Cerebellum, Basal Ganglia, and Cortex. Cerebellum. 2017;16:203–29. 26873754 10.1007/s12311-016-0763-3 PMC5243918

[74] Stoodley CJ, Valera EM, Schmahmann JD. Functional topography of the cerebellum for motor and cognitive tasks: an fMRI study. NeuroImage. 2012;59:1560–70. 21907811 10.1016/j.neuroimage.2011.08.065 PMC3230671

[75] Hoche F, Guell X, Sherman JC, Vangel MG, Schmahmann JD. Cerebellar Contribution to Social Cognition. The Cerebellum. 2015. 10.1007/s12311-015-0746-9 PMC5157127 26585120

[76] Schmahmann JD. Disorders of the cerebellum: ataxia, dysmetria of thought, and the cerebellar cognitive affective syndrome. J Neuropsychiatry Clin Neurosci. 2004;16:367–78. 15377747 10.1176/jnp.16.3.367

[77] Schmahmann JD, Sherman JC. Cerebellar cognitive affective syndrome. Int Rev Neurobiol. 1997;41:433–40. 9378601 10.1016/s0074-7742(08)60363-3

[78] Tavano A, Grasso R, Gagliardi C, Triulzi F, Bresolin N, Fabbro F, et al. Disorders of cognitive and affective development in cerebellar malformations. Brain. 2007;130:2646–60. 17872929 10.1093/brain/awm201

[79] Rogers TD, Dickson PE, Heck DH, Goldowitz D, Mittleman G, Blaha CD. Connecting the dots of the cerebro-cerebellar role in cognitive function: neuronal pathways for cerebellar modulation of dopamine release in the prefrontal cortex. Synapse. 2011;65:1204–12. 21638338 10.1002/syn.20960 PMC3854794

[80] Brooks SP, Trueman RC, Dunnett SB. Assessment of Motor Coordination and Balance in Mice Using the Rotarod, Elevated Bridge, and Footprint Tests. Curr Protoc Mouse Biol. 2012;2:37–53. 26069004 10.1002/9780470942390.mo110165

[81] Wertman V, Gromova A, La Spada AR, Cortes CJ. Low-Cost Gait Analysis for Behavioral Phenotyping of Mouse Models of Neuromuscular Disease. JoVE (Journal Visualized Experiments). 2019;2019:e59878. 10.3791/59878 PMC7553151 31380846

[82] Dixit PV, Sahu R, Mishra DK. Marble-burying behavior test as a murine model of compulsive-like behavior. J Pharmacol Toxicol Methods. 2020;102:106676. 31954839 10.1016/j.vascn.2020.106676

[83] Thomas A, Burant A, Bui N, Graham D, Yuva-Paylor LA, Paylor R. Marble burying reflects a repetitive and perseverative behavior more than novelty-induced anxiety. Psychopharmacology. 2009;204:361–73. 19189082 10.1007/s00213-009-1466-y PMC2899706

[84] Kraeuter AK, Guest PC, Sarnyai Z. The Y-Maze for Assessment of Spatial Working and Reference Memory in Mice. Methods Mol Biol. 2019;1916:105–11. 30535688 10.1007/978-1-4939-8994-2_10

[85] Rein B, Ma K, Yan Z. A standardized social preference protocol for measuring social deficits in mouse models of autism. Nat Protoc. 2020;15:3464–77. 32895524 10.1038/s41596-020-0382-9 PMC8103520

[86] Manders EMM, Verbeek FJ, Aten JA. Measurement of co-localization of objects in dual-colour confocal images. J Microsc. 1993;169:375–82. 33930978 10.1111/j.1365-2818.1993.tb03313.x

[87] Chabrol FP, Arenz A, Wiechert MT, Margrie TW, DiGregorio DA. Synaptic diversity enables temporal coding of coincident multisensory inputs in single neurons. Nat Neurosci. 2015;18:718–27. 25821914 10.1038/nn.3974 PMC4413433

[88] Richardson BD, Rossi DJ. Recreational concentrations of alcohol enhance synaptic inhibition of cerebellar unipolar brush cells via pre- and postsynaptic mechanisms. J Neurophysiol. 2017;118:267–79. 28381493 10.1152/jn.00963.2016 PMC5498730

[89] Kaplan JS, Nipper MA, Richardson BD, Jensen J, Helms M, Finn DA, et al. Pharmacologically Counteracting a Phenotypic Difference in Cerebellar GABAA Receptor Response to Alcohol Prevents Excessive Alcohol Consumption in a High Alcohol-Consuming Rodent Genotype. J Neurosci. 2016;36:9019. 27581446 10.1523/JNEUROSCI.0042-16.2016 PMC5005716

[90] Gebre SA, Reeber SL, Sillitoe RV. Parasagittal compartmentation of cerebellar mossy fibers as revealed by the patterned expression of vesicular glutamate transporters VGLUT1 and VGLUT2. Brain Struct Funct. 2012;217:165–80. 21814870 10.1007/s00429-011-0339-4

[91] Cathala L, Brickley S, Cull-Candy S, Farrant M. Maturation of EPSCs and Intrinsic Membrane Properties Enhances Precision at a Cerebellar Synapse. J Neurosci. 2003;23:6074–85. 12853426 10.1523/JNEUROSCI.23-14-06074.2003 PMC6740347

[92] Sathyanesan A, Zhou J, Scafidi J, Heck DH, Sillitoe RV, Gallo V. Emerging connections between cerebellar development, behaviour and complex brain disorders. Nat Rev Neurosci. 2019;20:298–313. 30923348 10.1038/s41583-019-0152-2 PMC7236620

[93] Osman A, Mervosh NL, Strat AN, Euston TJ, Zipursky G, Pollak RM, et al. Acetate supplementation rescues social deficits and alters transcriptional regulation in prefrontal cortex of Shank3 deficient mice. Brain Behav Immun. 2023;114:311–24. 37657643 10.1016/j.bbi.2023.08.020 PMC10955506

[94] Zhou Y, Kaiser T, Monteiro P, Van der Zhang X, Wang D, et al. Mice with Shank3 Mutations Associated with ASD and Schizophrenia Display Both Shared and Distinct Defects. Neuron. 2016;89:147–62. 26687841 10.1016/j.neuron.2015.11.023 PMC4754122

[95] Kouser M, Speed HE, Dewey CM, Reimers JM, Widman AJ, Gupta N, et al. Loss of predominant Shank3 isoforms results in hippocampus-dependent impairments in behavior and synaptic transmission. J Neurosci. 2013;33:18448–68. 24259569 10.1523/JNEUROSCI.3017-13.2013 PMC3834052

[96] Furuichi T, Shiraishi-Yamaguchi Y, Sato A, Sadakata T, Huang J, Shinoda Y, et al. Systematizing and cloning of genes involved in the cerebellar cortex circuit development. Neurochem Res. 2011;36:1241–52. 21243430 10.1007/s11064-011-0398-1

[97] Sato A, Sekine Y, Saruta C, Nishibe H, Morita N, Sato Y, et al. Cerebellar development transcriptome database (CDT-DB): profiling of spatio-temporal gene expression during the postnatal development of mouse cerebellum. Neural Netw. 2008;21:1056–69. 18603407 10.1016/j.neunet.2008.05.004

[98] Ziats CA, Grosvenor LP, Sarasua SM, Thurm AE, Swedo SE, Mahfouz A, et al. Functional genomics analysis of Phelan-McDermid syndrome 22q13 region during human neurodevelopment. PLoS ONE. 2019;1:13. 10.1371/journal.pone.0213921 PMC6420160 30875393

[99] D’Mello AM, Crocetti D, Mostofsky SH, Stoodley CJ. Cerebellar gray matter and lobular volumes correlate with core autism symptoms. Neuroimage Clin. 2015;7:631–9. 25844317 10.1016/j.nicl.2015.02.007 PMC4375648

[100] Stoodley CJ. Distinct regions of the cerebellum show gray matter decreases in autism, ADHD, and developmental dyslexia. Front Syst Neurosci. 2014;8:92. 24904314 10.3389/fnsys.2014.00092 PMC4033133

[101] Wegiel J, Kuchna I, Nowicki K, Imaki H, Wegiel J, Marchi E, et al. The neuropathology of autism: Defects of neurogenesis and neuronal migration, and dysplastic changes. Acta Neuropathol. 2010;119:755–70. 20198484 10.1007/s00401-010-0655-4 PMC2869041

[102] Hashimoto T, Tayama M, Murakawa K, Yoshimoto T, Miyazaki M, Harada M, et al. Development of the brainstem and cerebellum in autistic patients. J Autism Dev Disord. 1995;25:1–18. 7608030 10.1007/BF02178163

[103] Courchesne E, Karns CM, Davis HR, Ziccardi R, Carper RA, Tigue ZD, et al. Unusual brain growth patterns in early life in patients with autistic disorder: an MRI study. Neurology. 2001;57:245–54. 11468308 10.1212/wnl.57.2.245

[104] Skefos J, Cummings C, Enzer K, Holiday J, Weed K, Levy E, et al. Regional alterations in purkinje cell density in patients with autism. PLoS ONE. 2014;9:e81255. 24586223 10.1371/journal.pone.0081255 PMC3933333

[105] Limperopoulos C, Bassan H, Gauvreau K, Robertson RL, Sullivan NR, Benson CB, et al. Does cerebellar injury in premature infants contribute to the high prevalence of long-term cognitive, learning, and behavioral disability in survivors? Pediatrics. 2007;120:584–93. 17766532 10.1542/peds.2007-1041

[106] Murphy CM, Christakou A, Daly EM, Ecker C, Giampietro V, Brammer M, et al. Abnormal functional activation and maturation of fronto-striato-temporal and cerebellar regions during sustained attention in autism spectrum disorder. Am J Psychiatry. 2014;171:1107–16. 24873905 10.1176/appi.ajp.2014.12030352

[107] Brodski-Guerniero A, Naumer MJ, Moliadze V, Chan J, Althen H, Ferreira-Santos F, et al. Predictable information in neural signals during resting state is reduced in autism spectrum disorder. Hum Brain Mapp. 2018;1:14. 10.1002/hbm.24072 PMC6866422 29617056

[108] Zalla T, Seassau M, Cazalis F, Gras D, Leboyer M. Saccadic eye movements in adults with high-functioning autism spectrum disorder. Autism. 2018;22:195–204. 29490485 10.1177/1362361316667057

[109] Carson TB, Wilkes BJ, Patel K, Pineda JL, Ko JH, Newell KM, et al. Vestibulo-ocular reflex function in children with high-functioning autism spectrum disorders. Autism Res. 2017;10:251–66. 27220548 10.1002/aur.1642

[110] Cardon GJ, Hepburn S, Rojas DC. Structural Covariance of Sensory Networks, the Cerebellum, and Amygdala in Autism Spectrum Disorder. Front Neurol. 2017;8:615. 29230189 10.3389/fneur.2017.00615 PMC5712069

[111] Mostofsky SH, Powell SK, Simmonds DJ, Goldberg MC, Caffo B, Pekar JJ. Decreased connectivity and cerebellar activity in autism during motor task performance. Brain. 2009;132:2413–25. 19389870 10.1093/brain/awp088 PMC2732264

[112] Laidi C, Boisgontier J, Chakravarty MM, Hotier S, D’Albis M-A, Mangin J-F, et al. Cerebellar anatomical alterations and attention to eyes in autism. Sci Rep. 2017;7:12008. 28931838 10.1038/s41598-017-11883-w PMC5607223

