# Supplementary material for: Behavioral decline in Shank3Δex4–22 mice during early adulthood parallels cerebellar granule cell glutamatergic synaptic changes
Source: Mol Autism. 2024 Dec 4;15:52. doi: 10.1186/s13229-024-00628-y (PMC11616285; doi:10.1186/s13229-024-00628-y)
Supplement: Supplementary file 1 — Supplementary Material 1: Statistical results for all behavioral analyses. [file 13229_2024_628_MOESM1_ESM.pdf]

Assay	Levene's (Median) Homogeneity	Factorial Test	Grouping Variable	Multiple Comparisons																			N			Posthoc		
				KW Statistic (X^2)	KW p-Value	MANOVA Results	Repeated Factor (e.g trial)		Genotype		Age		Sex		Genotype * Age		Genotype*Sex		Age*Sex		Genotype*Age*Sex		WT	Het	KO	WT vs HT	WT vs KO	HT vs KO
							F	p-value	F	p-value	F	p-value	F	p-value	F	p-value	F	p-value	F	p-value	F	p-value						
OF - Center	**0.010**	Kruskal	All Combined	47.648	**0.000**																		56	56	50	**0.002**	**0.000**	**0.001**
Entry Count		Wallis H	Adult	27.404	**0.000**																		30	27	25	0.101	**0.000**	**0.006**
			Juvenile	20.560	**0.000**																		26	29	25	**0.015**	**0.000**	0.199
			Adult Male	16.077	**0.000**																		14	13	12	0.392	**0.000**	**0.041**
			Juvenile Male	11.136	**0.004**																		15	15	13	0.078	**0.003**	0.805
			Adult Female	11.758	**0.003**																		16	14	13	0.359	**0.002**	0.195
			Juvenile Female	10.031	**0.007**																		11	14	12	0.233	**0.005**	0.361
OF - Center	0.102	3-way	All Combined						14.270	**0.000**	13.335	**0.000**	3.282	0.072	1.609	0.203	1.769	0.174	0.003	0.957	0.098	0.907	56	56	50	**0.005**	**0.000**	0.085
Time		ANOVA	Adult																				30	27	25	**0.035**	**0.000**	**0.041**
			Juvenile																				26	29	25	0.155	**0.042**	1.000
			Adult Male																				14	13	12	0.126	**0.000**	0.097
			Juvenile Male																				15	15	13	0.205	**0.009**	0.637
			Adult Female																				16	14	13	0.375	**0.012**	0.526
			Juvenile Female																				11	14	12	0.982	1.000	1.000
OF - Freezing	**0.006**	Kruskal	All Combined	0.916	0.633																		56	56	50			
Time		Wallis H	Adult	2.419	0.298																		30	27	25			
			Juvenile	2.379	0.304																		26	29	25			
			Adult Male	2.866	0.239																		14	13	12			
			Juvenile Male	4.791	0.091																		15	15	13			
			Adult Female	0.261	0.878																		16	14	13			
			Juvenile Female	0.162	0.922																		11	14	12			
OF - Fecal Boli	0.370	3-way	All Combined						0.632	0.533	33.847	**0.000**	10.619	**0.001**	2.103	0.126	1.081	0.342	9.662	**0.002**	0.844	0.432	56	56	50			
		ANOVA	Adult																				30	27	25			
			Juvenile																				26	29	25			
			Adult Male																				14	13	12			
			Juvenile Male																				15	15	13			
			Adult Female																				16	14	13			
			Juvenile Female																				11	14	12			
Zero Maze -	**0.000**	Kruskal	All Combined	42.418	**0.000**																		51	50	53	1.000	**0.000**	**0.000**
Open Arm		Wallis H	Adult	38.495	**0.000**																		30	28	30	0.288	**0.000**	**0.000**
Time			Juvenile	13.157	**0.001**																		21	22	23	0.510	0.091	**0.001**
			Adult Male	19.597	**0.000**																		14	14	15	0.061	**0.000**	0.116
			Juvenile Male	7.385	**0.025**																		11	12	12	0.990	0.293	**0.021**
			Adult Female	21.175	**0.000**																		16	14	15	1.000	**0.000**	**0.000**
			Juvenile Female	5.135	0.077																		10	10	11	1.000	0.586	0.073
Zero Maze -	0.425	3-way	All Combined						10.891	**0.000**	6.109	**0.015**	22.359	**0.000**	0.580	0.561	0.101	0.904	6.182	**0.014**	1.005	0.369	51	50	53	1.000	**0.002**	**0.000**
Open Arm		ANOVA	Adult																				30	28	30	1.000	**0.002**	**0.002**
Entries			Juvenile																				21	22	23	0.850	0.310	**0.020**
			Adult Male																				14	14	15	1.000	**0.010**	0.083
			Juvenile Male																				11	12	12	0.608	1.000	0.217
			Adult Female																				16	14	15	1.000	0.152	**0.021**
			Juvenile Female																				10	10	11	1.000	0.223	0.117
OF -	0.787	3-way	All Combined			Between Subjects Cumm Dis			28.378	**0.000**	3.931	**0.049**	8.222	**0.005**	0.400	0.671	0.253	0.777	0.126	0.723	0.301	0.740	56	56	50	**0.009**	**0.000**	**0.000**
Cummulative		ANOVA	Adult	Box's Test		Within Subjects (x Time)	227.345	**0.000**	3.441	**0.000**	2.152	0.066	2.096	0.073	0.902	0.521	1.014	0.427	1.507	0.193	1.123	0.344	30	27	25	**0.020**	**0.000**	**0.007**
Distance			Juvenile	M	p-value	Between Subjects MANOVA			28.218	**0.000**	4.019	**0.047**	8.354	**0.004**	0.392	0.676	0.240	0.787	0.112	0.738	0.285	0.753	26	29	25	0.396	**0.000**	**0.003**
			Adult Male	374.396	**0.002**																		14	13	12	0.052	**0.000**	0.420
			Juvenile Male			Mauchly's Test																	15	15	13	0.756	**0.001**	**0.043**
			Adult Female	W	p-value	Huynh-Feldt Epsilon																	16	14	13	0.443	**0.000**	**0.011**
			Juvenile Female	0.503	**0.000**	0.885																	11	14	12	0.955	**0.006**	0.070
OF - Distance -		MANOVA	All Combined																				56	56	50	1.000	**0.001**	**0.001**
0-5min			Adult																				30	27	25	1.000	**0.034**	0.157
			Juvenile																				26	29	25	1.000	**0.003**	**0.005**
			Adult Male																				14	13	12	1.000	0.735	0.919
			Juvenile Male																				15	15	13	0.836	**0.012**	0.182
			Adult Female																				16	14	13	1.000	**0.041**	0.245
			Juvenile Female																				11	14	12	1.000	0.186	**0.030**
OF - Distance -		MANOVA	All Combined																				56	56	50	**0.047**	**0.000**	**0.000**
5-10min			Adult																				30	27	25	0.103	**0.000**	**0.000**
			Juvenile																				26	29	25	0.560	**0.000**	**0.001**
			Adult Male																				14	13	12	0.421	**0.000**	0.054
			Juvenile Male																				15	15	13	0.430	**0.000**	**0.047**
			Adult Female																				16	14	13	0.378	**0.000**	**0.000**
			Juvenile Female																				11	14	12	1.000	**0.004**	**0.009**
OF - Distance -		MANOVA	All Combined																				56	56	50	**0.015**	**0.000**	**0.000**
10-15min			Adult																				30	27	25	**0.048**	**0.000**	**0.002**
			Juvenile																				26	29	25	0.344	**0.001**	0.063
			Adult Male																				14	13	12	**0.008**	**0.000**	0.260
			Juvenile Male																				15	15	13	1.000	**0.013**	0.103
			Adult Female																				16	14	13	1.000	**0.001**	**0.005**
			Juvenile Female																				11	14	12	0.470	**0.042**	0.718
OF - Distance -		MANOVA	All Combined																				56	56	50	**0.001**	**0.000**	**0.005**
15-20min			Adult																				30	27	25	**0.004**	**0.000**	**0.034**
			Juvenile																				26	29	25	0.087	**0.000**	0.143
			Adult Male																				14	13	12	**0.027**	**0.001**	0.905
			Juvenile Male																				15	15	13	0.375	**0.032**	0.814
			Adult Female																				16	14	13	0.172	**0.000**	**0.029**
			Juvenile Female																				11	14	12	0.351	**0.006**	0.268
OF - Distance -		MANOVA	All Combined																				56	56	50	0.573	**0.000**	**0.002**

20-25min			Adult																				30	27	25	0.491	**0.001**	0.079
			Juvenile																				26	29	25	1.000	**0.010**	**0.030**
			Adult Male																				14	13	12	0.603	**0.006**	0.199
			Juvenile Male																				15	15	13	1.000	**0.035**	**0.020**
			Adult Female																				16	14	13	1.000	0.136	0.577
			Juvenile Female																				11	14	12	1.000	0.269	1.000
OF - Distance -		MANOVA	All Combined																				56	56	50	**0.010**	**0.000**	0.770
25-30min			Adult																				30	27	25	**0.009**	**0.012**	1.000
			Juvenile																				26	29	25	0.648	**0.017**	0.298
			Adult Male																				14	13	12	**0.028**	0.360	0.954
			Juvenile Male																				15	15	13	1.000	0.202	0.762
			Adult Female																				16	14	13	0.333	**0.031**	0.966
			Juvenile Female																				11	14	12	0.927	0.108	0.698
OF - Max	0.240	3-way	All Combined						1.277	0.282	4.069	**0.045**	3.940	**0.049**	0.304	0.738	1.919	0.150	3.428	0.066	0.650	0.523	56	56	50			
Velocity		ANOVA	Adult																				30	27	25			
			Juvenile																				26	29	25			
			Adult Male																				14	13	12			
			Juvenile Male																				15	15	13			
			Adult Female																				16	14	13			
			Juvenile Female																				11	14	12			
Rotarod - Spin -		MANOVA	All Combined																				53	49	49	1.000	**0.040**	**0.036**
T1			Adult	Box's Test		Within Subjects (x Time)	45.427	0.000	1.671	0.086	1.584	0.164	0.778	0.562	1.401	0.178	1.508	0.135	3.692	**0.003**	0.647	0.769	30	23	24	1.000	**0.043**	0.070
			Juvenile	M	p-value	Between Subjects MANOVA			20.872	**0.000**	23.805	**0.000**	12.854	**0.000**	1.900	0.153	0.523	0.594	0.414	0.521	0.181	0.834	23	26	25	1.000	0.816	0.605
			Adult Male	371.266	**0.005**																		16	12	10	1.000	0.133	0.330
			Juvenile Male	Mauchly's Test																			12	13	13	1.000	0.509	1.000
			Adult Female	W	p-value	Huynh-Feldt Epsilon																	14	11	14	1.000	0.441	0.313
			Juvenile Female	0.722	**0.000**	0.971																	11	13	12	1.000	1.000	0.944
Rotarod - Spin -		3-way	All Combined																				53	49	49	0.875	**0.000**	**0.000**
T2		ANOVA	Adult																				30	23	24	1.000	**0.000**	**0.004**
			Juvenile																				23	26	25	1.000	**0.006**	**0.035**
			Adult Male																				16	12	10	1.000	0.089	0.091
			Juvenile Male																				12	13	13	1.000	0.097	0.331
			Adult Female																				14	11	14	0.676	**0.000**	**0.042**
			Juvenile Female																				11	13	12	1.000	0.065	0.142
Rotarod - Spin -		3-way	All Combined																				53	49	49	0.733	**0.000**	**0.000**
T3		ANOVA	Adult																				30	23	24	0.111	**0.000**	**0.007**
			Juvenile																				23	26	25	1.000	**0.000**	**0.000**
			Adult Male																				16	12	10	0.265	**0.001**	0.144
			Juvenile Male																				12	13	13	0.560	0.173	**0.004**
			Adult Female																				14	11	14	0.615	**0.000**	0.055
			Juvenile Female																				11	13	12	1.000	**0.000**	**0.002**
Rotarod - Spin -		3-way																					53	49	49	0.211	**0.000**	**0.016**
T4		ANOVA	Adult																				30	23	24	0.085	**0.000**	0.159
			Juvenile																				23	26	25	1.000	0.055	0.123
			Adult Male																				16	12	10	1.000	**0.008**	**0.022**
			Juvenile Male																				12	13	13	1.000	0.198	0.344
			Adult Female																				14	11	14	**0.013**	**0.009**	1.000
			Juvenile Female																				11	13	12	1.000	0.383	0.556
Rotarod - Spin -		3-way																					53	49	49	0.690	**0.002**	0.075
T5		ANOVA	Adult																				30	23	24	0.202	**0.000**	0.090
			Juvenile																				23	26	25	1.000	1.000	0.989
			Adult Male																				16	12	10	0.936	**0.025**	0.326
			Juvenile Male																				12	13	13	1.000	1.000	1.000
			Adult Female																				14	11	14	0.350	**0.004**	0.418
			Juvenile Female																				11	13	12	1.000	1.000	1.000
Rotarod - Spin -		3-way	All Combined																				53	49	49	0.228	**0.000**	0.065
T6		ANOVA	Adult																				30	23	24	0.573	**0.000**	**0.010**
			Juvenile																				23	26	25	0.678	0.449	1.000
			Adult Male																				16	12	10	1.000	**0.003**	**0.005**
			Juvenile Male																				12	13	13	1.000	1.000	0.960
			Adult Female																				14	11	14	0.204	**0.012**	1.000
			Juvenile Female																				11	13	12	0.246	0.794	1.000
Rotarod - Fall -		MANOVA	All Combined																				53	49	49	0.926	**0.000**	**0.001**
T1			Adult	Box's Test		Within Subjects (x Time)	40.560	**0.000**	0.622	0.794	1.683	0.137	0.927	0.462	1.434	0.162	0.795	0.633	0.827	0.530	0.779	0.648	30	23	24	1.000	**0.004**	**0.029**
			Juvenile	M	p-value	Between Subjects MANOVA			33.381	**0.000**	32.791	**0.000**	15.655	**0.000**	3.374	**0.037**	0.131	0.877	2.589	0.110	0.782	0.459	23	26	25	1.000	**0.004**	0.051
			Adult Male	330.180	0.108																		16	12	10	0.957	**0.010**	0.172
			Juvenile Male	Mauchly's Test																			12	13	13	1.000	0.107	0.293
			Adult Female	W	p-value	Huynh-Feldt Epsilon																	14	11	14	1.000	0.296	0.225
			Juvenile Female	0.746	**0.000**	0.991																	11	13	12	1.000	**0.043**	0.246
Rotarod - Fall -		MANOVA	All Combined																				53	49	49	1.000	**0.000**	**0.000**
T2			Adult																				30	23	24	0.396	**0.000**	**0.001**
			Juvenile																				23	26	25	1.000	0.265	**0.044**
			Adult Male																				16	12	10	0.438	**0.000**	**0.018**
			Juvenile Male																				12	13	13	0.565	1.000	0.163
			Adult Female																				14	11	14	1.000	**0.002**	**0.037**
			Juvenile Female																				11	13	12	1.000	0.212	0.361
Rotarod - Fall -		MANOVA	All Combined																				53	49	49	0.514	**0.000**	**0.000**
T3			Adult																				30	23	24	0.705	**0.000**	**0.000**
			Juvenile																				23	26	25	1.000	**0.008**	0.055
			Adult Male																				16	12	10	1.000	**0.002**	**0.015**
			Juvenile Male																				12	13	13	1.000	0.662	0.399
			Adult Female																				14	11	14	0.688	**0.000**	**0.003**

			Juvenile Female																	11	13	12	0.581	**0.008**	0.195
Rotarod - Fall -		MANOVA	All Combined																	53	49	49	**0.028**	**0.000**	**0.028**
T4			Adult																	30	23	24	**0.017**	**0.000**	0.156
			Juvenile																	23	26	25	1.000	**0.027**	0.238
			Adult Male																	16	12	10	0.101	**0.000**	0.059
			Juvenile Male																	12	13	13	0.528	0.245	1.000
			Adult Female																	14	11	14	0.202	0.063	1.000
			Juvenile Female																	11	13	12	1.000	0.148	0.117
Rotarod - Fall -		MANOVA	All Combined																	53	49	49	0.612	**0.000**	**0.002**
T5			Adult																	30	23	24	0.251	**0.000**	**0.006**
			Juvenile																	23	26	25	1.000	0.267	0.287
			Adult Male																	16	12	10	0.226	**0.000**	**0.041**
			Juvenile Male																	12	13	13	1.000	1.000	1.000
			Adult Female																	14	11	14	1.000	**0.018**	0.171
			Juvenile Female																	11	13	12	1.000	0.316	0.425
Rotarod - Fall -		MANOVA	All Combined																	53	49	49	0.058	**0.000**	**0.038**
T6			Adult																	30	23	24	1.000	**0.000**	**0.001**
			Juvenile																	23	26	25	**0.039**	0.099	1.000
			Adult Male																	16	12	10	1.000	**0.002**	**0.009**
			Juvenile Male																	12	13	13	0.507	0.086	1.000
			Adult Female																	14	11	14	1.000	**0.002**	0.061
			Juvenile Female																	11	13	12	0.097	1.000	0.579
Beam Balance -	**0.041**	Kruskal	All Combined	4.149	0.126															52	51	56	1.000	0.307	0.192
6mm - Time		Wallis H	Adult	0.553	0.758															32	26	35	1.000	1.000	1.000
			Juvenile	10.729	**0.005**															20	25	21	1.000	**0.007**	**0.029**
			Adult Male	1.827	0.401															17	13	17	0.538	1.000	1.000
			Juvenile Male	5.050	0.080															10	14	11	1.000	0.665	0.074
			Adult Female	0.601	0.740															15	13	18	1.000	1.000	1.000
			Juvenile Female	8.898	**0.012**															10	11	10	0.535	**0.009**	0.267
Beam Balance -	**0.028**	Kruskal	All Combined	13.788	**0.001**															52	51	56	0.771	**0.001**	**0.043**
6mm - Slips		Wallis H	Adult	10.086	**0.006**															32	26	35	1.000	**0.006**	0.126
			Juvenile	4.281	0.118															20	25	21	1.000	0.117	0.691
			Adult Male	5.529	0.063															17	13	17	0.234	0.084	1.000
			Juvenile Male	2.869	0.238															10	14	11	1.000	0.271	1.000
			Adult Female	9.499	**0.009**															15	13	18	1.000	0.081	**0.011**
			Juvenile Female	1.562	0.458															10	11	10	1.000	0.700	1.000
Beam Balance -	**0.001**	Kruskal	All Combined	28.511	**0.000**															52	51	56	1.000	**0.000**	**0.000**
12mm - Time		Wallis H	Adult	12.963	**0.002**															32	26	35	1.000	**0.005**	**0.009**
			Juvenile	16.241	**0.000**															20	25	21	0.839	**0.000**	**0.009**
			Adult Male	11.703	**0.003**															17	13	17	1.000	**0.007**	**0.015**
			Juvenile Male	11.949	**0.003**															10	14	11	1.000	**0.004**	**0.019**
			Adult Female	2.451	0.294															15	13	18	1.000	0.641	0.486
			Juvenile Female	5.323	0.070															10	11	10	0.544	0.064	0.927
Beam Balance -	**0.006**	Kruskal	All Combined	7.538	**0.023**															52	51	56	1.000	**0.041**	0.076
12mm - Slips		Wallis H	Adult	9.790	**0.007**															32	26	35	1.000	0.090	**0.008**
			Juvenile	3.094	0.213															20	25	21	0.252	0.690	1.000
			Adult Male	4.716	0.095															17	13	17	0.570	1.000	0.090
			Juvenile Male	5.541	0.063															10	14	11	0.079	1.000	0.291
			Adult Female	6.334	**0.042**															15	13	18	1.000	0.078	0.128
			Juvenile Female	2.035	0.362															10	11	10	1.000	0.695	0.602
Gait - Forelimb	0.416	3-way	All Combined			7.548	**0.001**	30.609	**0.000**	0.074	0.787	4.838	**0.009**	2.551	0.082	0.548	0.460	2.178	0.118	44	45	46	1.000	**0.001**	**0.009**
- Length		ANOVA	Adult																	24	23	25	0.213	0.206	1.000
			Juvenile																	20	22	21	1.000	**0.005**	**0.000**
			Adult Male																	14	10	11	1.000	0.676	0.368
			Juvenile Male																	10	12	11	1.000	**0.004**	**0.001**
			Adult Female																	10	13	14	**0.010**	0.514	0.222
			Juvenile Female																	10	10	10	0.928	0.609	0.069
Gait - Hindlimb	0.448	3-way	All Combined			8.112	**0.000**	29.755	**0.000**	0.111	0.739	4.973	**0.008**	2.217	0.113	0.352	0.554	2.224	0.113	44	45	46	1.000	**0.001**	**0.007**
- Length		ANOVA	Adult																	24	23	25	0.149	0.132	1.000
			Juvenile																	20	22	21	1.000	**0.005**	**0.000**
			Adult Male																	14	10	11	1.000	0.592	0.451
			Juvenile Male																	10	12	11	1.000	**0.003**	**0.001**
			Adult Female																	10	13	14	**0.009**	0.351	0.318
			Juvenile Female																	10	10	10	0.794	0.661	0.061
Gait - Forelimb	0.642	3-way	All Combined			11.878	**0.000**	17.626	**0.000**	6.295	**0.013**	1.504	0.226	0.689	0.504	0.011	0.915	4.920	**0.009**	44	45	46	0.485	**0.004**	**0.000**
- Width		ANOVA	Adult																	24	23	25	0.315	**0.008**	**0.000**
			Juvenile																	20	22	21	1.000	0.304	0.119
			Adult Male																	14	10	11	1.000	0.084	0.271
			Juvenile Male																	10	12	11	1.000	0.086	**0.006**
			Adult Female																	10	13	14	**0.027**	0.108	**0.000**
			Juvenile Female																	10	10	10	1.000	1.000	1.000
Gait - Hindlimb	0.312	3-way	All Combined			0.980	0.378	1.493	0.224	8.154	**0.005**	2.184	0.117	0.770	0.465	0.021	0.885	0.325	0.723	44	45	46	1.000	0.740	0.641
- Width		ANOVA	Adult																	24	23	25	0.514	0.133	1.000
			Juvenile																	20	22	21	0.476	1.000	0.775
			Adult Male																	14	10	11	1.000	1.000	1.000
			Juvenile Male																	10	12	11	0.410	1.000	0.609
			Adult Female																	10	13	14	0.268	0.076	1.000
			Juvenile Female																	10	10	10	1.000	1.000	1.000
OF - Grooming	0.440	3-way	All Combined			8.648	**0.000**	0.004	0.950	6.696	**0.011**	1.286	0.279	4.067	**0.019**	1.611	0.206	0.648	0.524	56	56	50	0.082	**0.000**	0.144
Duration		ANOVA	Adult																	30	27	25	0.130	0.071	1.000
			Juvenile																	26	29	25	0.784	**0.001**	**0.035**
			Adult Male																	14	13	12	**0.008**	**0.013**	1.000

			Juvenile Male																	15	15	13	0.403	**0.000**	**0.042**
			Adult Female																	16	14	13	1.000	1.000	1.000
			Juvenile Female																	11	14	12	1.000	0.649	0.748
Marbles Burried - 30 min	**0.001**	Kruskal Wallis H		39.379	**0.000**															48	49	51	1.000	**0.000**	**0.000**
			Adult	27.991	**0.000**															28	26	30	0.364	**0.000**	**0.001**
			Juvenile	20.733	**0.000**															20	23	21	0.893	**0.004**	**0.000**
			Adult Male	18.814	**0.000**															15	13	17	1.000	**0.000**	**0.008**
			Juvenile Male	8.556	**0.014**															10	12	11	1.000	0.096	**0.016**
			Adult Female	9.126	**0.010**															13	13	13	0.646	**0.008**	0.232
			Juvenile Female	11.381	**0.003**															10	11	10	1.000	0.061	**0.003**
Y-Maze - Total Entries	0.305	3-way ANOVA	All Combined			19.606	**0.000**	0.369	0.544	15.698	**0.000**	2.894	0.058	0.671	0.513	0.167	0.683	0.836	0.435	54	58	54	0.232	**0.000**	**0.000**
			Adult																	30	27	30	0.112	**0.001**	0.465
			Juvenile																	24	31	24	1.000	**0.000**	**0.000**
			Adult Male																	14	13	15	0.847	0.585	1.000
			Juvenile Male																	12	18	12	1.000	**0.002**	**0.001**
			Adult Female																	16	14	15	0.173	**0.000**	0.195
			Juvenile Female																	12	13	12	1.000	**0.002**	**0.009**
Y-Maze - Alternation %	0.959	3-way ANOVA	All Combined			2.133	0.122	0.310	0.578	1.081	0.300	0.360	0.698	0.640	0.528	1.365	0.244	1.669	0.192	54	58	54			
			Adult																	30	27	30			
			Juvenile																	24	31	24			
			Adult Male																	14	13	15			
			Juvenile Male																	12	18	12			
			Adult Female																	16	14	15			
			Juvenile Female																	12	13	12			
Social Preference Index	0.246	3-way ANOVA	All Combined			1.770	0.174	3.081	0.081	2.203	0.140	3.676	**0.028**	0.600	0.550	0.325	0.570	0.043	0.957	56	53	52	1.000	0.279	0.347
			Adult																	33	25	30	1.000	**0.004**	**0.023**
			Juvenile																	23	28	22	1.000	1.000	1.000
			Adult Male																	16	14	16	1.000	**0.025**	0.181
			Juvenile Male																	12	15	9	1.000	1.000	1.000
			Adult Female																	17	11	14	1.000	0.146	0.166
			Juvenile Female																	11	13	13	1.000	1.000	1.000
Social Novelty Index	0.098	3-way ANOVA	All Combined			0.287	0.751	5.556	**0.020**	0.336	0.563	0.822	0.442	0.359	0.699	2.486	0.117	1.104	0.335	53	48	48	1.000	1.000	1.000
			Adult																	31	25	28	1.000	1.000	1.000
			Juvenile																	22	23	20	1.000	0.707	1.000
			Adult Male																	14	14	15	1.000	1.000	1.000
			Juvenile Male																	12	12	9	1.000	1.000	1.000
			Adult Female																	17	11	13	1.000	1.000	1.000
			Juvenile Female																	10	11	11	1.000	1.000	1.000
